# Electrochemical Switching of a Fluorescent Molecular Rotor Embedded within a Bistable Rotaxane

**DOI:** 10.1021/jacs.0c03701

**Published:** 2020-05-29

**Authors:** Yilei Wu, Marco Frasconi, Wei-Guang Liu, Ryan M. Young, William A. Goddard, Michael R. Wasielewski, J. Fraser Stoddart

**Affiliations:** ^†^Department of Chemistry and ^∥^Institute for Sustainability and Energy at Northwestern (ISEN), Northwestern University, 2145 Sheridan Road, Evanston, Illinois 60208, United States; ‡ Department of Chemical Sciences, University of Padova, Via Marzolo 1, Padova 35131, Italy; § Materials and Process Simulation Center, California Institute of Technology, Pasadena, California 91125, United States; ⊥ Institute for Molecular Design and Synthesis, Tianjin University, 92 Weijin Road, Nankai District, Tianjin 300072, China; # School of Chemistry, University of New South Wales, Sydney, New South Wales 2052, Australia

## Abstract

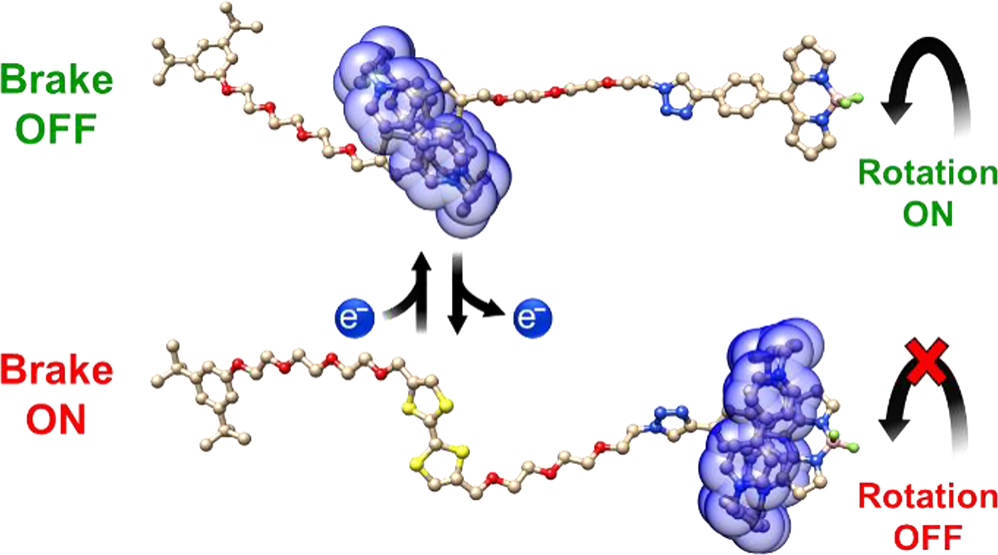

We report how the nanoconfined environment, introduced by the mechanical bonds within an electrochemically switchable bistable [2]rotaxane, controls the rotation of a fluorescent molecular rotor, namely, an 8-phenyl-substituted boron dipyrromethene (BODIPY). The electrochemical switching of the bistable [2]rotaxane induces changes in the ground-state coconformation and in the corresponding excited-state properties of the BODIPY rotor. In the starting redox state, when no external potential is applied, the cyclobis(paraquat-*p*-phenylene) (CBPQT^4+^) ring component encircles the tetrathiafulvalene (TTF) unit on the dumbbell component, leaving the BODIPY rotor unhindered and exhibiting low fluorescence. Upon oxidation of the TTF unit to a TTF^2+^ dication, the CBPQT^4+^ ring is forced toward the molecular rotor, leading to an increased energy barrier for the excited state to rotate the rotor into the state with a high nonradiative rate constant, resulting in an overall 3.4-fold fluorescence enhancement. On the other hand, when the solvent polarity is high enough to stabilize the excited charge-transfer state between the BODIPY rotor and the CBPQT^4+^ ring, movement of the ring toward the BODIPY rotor produces an unexpectedly strong fluorescence signal decrease as the result of photoinduced electron transfer from the BODIPY rotor to the CBPQT^4+^ ring. The nanoconfinement effect introduced by mechanical bonding can effectively lead to modulation of the physicochemical properties as observed in this bistable [2]rotaxane. On account of the straightforward synthetic strategy and the facile modulation of switchable electrochromic behavior, our approach could pave the way for the development of new stimuli-responsive materials based on mechanically interlocked molecules for future electro-optical applications, such as sensors, molecular memories, and molecular logic gates.

## Introduction

Molecular devices and machines are of considerable interest on account of their potential use in sensing, catalytic, electronic, and nanotechnological applications.^1^ Mechanically interlocked molecules (MIMs) constitute promising nanoscale molecular assemblies for the development of switches,^2^ actuators,^3^ ratchets,^4^ and motors.^5^ Well-known examples are bistable [2]rotaxanes,^2a,6^ which are MIMs comprising a ring component mechanically bonded onto a linear dumbbell component with two (or more) recognition sites for occupation by the ring component. The ability to manipulate reversibly the relative positioning of the ring component with respect to the dumbbell component is crucial to exploring their use in operating molecular devices. Controlled rotary movement of a ring component around a dumbbell has been achieved^7^ by introducing suitable steric hindrance between the two components of the rotaxane. On the other hand, the net linear translation of the ring with respect to a constitutionally asymmetric axle has been demonstrated in molecular shuttles^4b,4c^ and pseudorotaxanes^8^ by exploiting ratchet mechanisms.^9^ Recently, a rotaxane-based molecular shuttle, combined with an overcrowded alkene rotary motor, has led to transformation of the unidirectional rotation of the molecular motor into a reciprocating shuttling motion,^10^ thus coupling rotary and translational movements in a MIM. Indeed, rotary motors are a class of molecular machines that display controlled rotation of one component with respect to the other around single^11^ or double^12^ bonds driven, for the most part, by either light or heat.

Fluorescent molecular rotors are compounds whose photoluminescence is modulated by segmental mobility (twisting), that is, the locally excited (LE) electronic state can relax either by (i) the radiative emission of a photon or by (ii) formation of a “dark state” that relaxes nonradiatively to the ground state (GS) on account of internal rotation.^13^ If the local environment around the fluorophore permits rapid rotation in the excited state, fast nonradiative decay processes can effectively quench its fluorescence. On the other hand, any environmental restriction to twisting in the excited state because of free volume, molecular crowding, or solvent viscosity slows down rotational relaxation, enhancing fluorescence efficiency from the LE state. This environmental sensitivity of fluorescent molecular rotors has been exploited^13^ extensively in biological applications to probe, in real time, local microviscosity in biofluids and biomembranes.

**Scheme 1:**
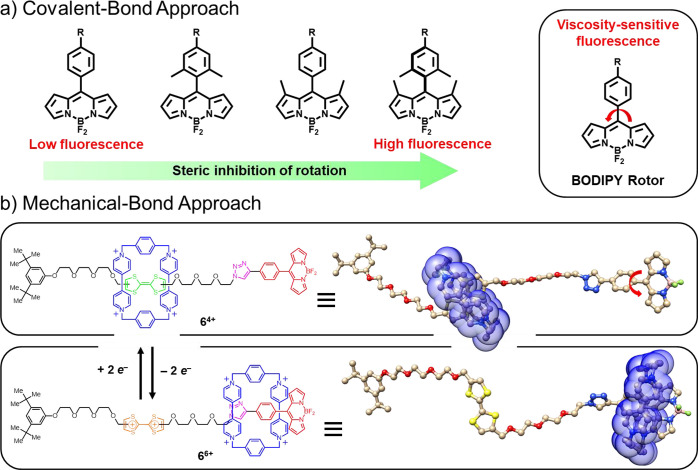
(a) Covalent-Bond Approach to Enhancing the Fluorescence of BODIPY by Introducing Steric Constraints on the Aryl Ring and Dipyrrin Core, and Structure of the BODIPY Rotor Employed as a Viscosity-Sensitive Probe; (b) Mechanical-Bond Approach to Controlling the Rotation of the BODIPY Rotor by Redox Actuation of a Bistable [2]Rotaxane **6**^4+^ Structural formulas and graphical representations of **6**^4+^ and its redox switching from **6**^4+^ to **6**^6+^.

One of the most widely used^14^ fluorescent molecular rotors is (Scheme 1a) the BODIPY rotor. Introduction of steric constraints in the form of substituents onto the phenyl ring or the dipyrrin units of the BODIPY is an effective approach to increasing the quantum yield of these rotors by preventing free rotation of the phenyl group, thus reducing loss of energy from the excited states via nonradiative molecular motions.^15^ Indeed, a theoretical study shows^15a^ that the phenyl ring rotation and accompanying boron-dipyrrin distortions allow access to an excited-state conformation with low radiative probability and facile nonradiative deactivation to the ground state, thereby limiting the fluorescence yields of the dyes. Such a distorted conformation is energetically inaccessible in a system bearing the sterically hindered *o*-tolyl or mesityl group, leading to a high radiative probability, and high fluorescence quantum yield, involving conformations at or near the initial Franck–Condon form of the excited state.^15b^ An increase in the fluorescence quantum yield of the BODIPY rotor is also observed^14^ by increasing the solvent viscosity, which results in restricted rotation of the phenyl group, thus preventing nonradiative relaxation. This property of the BODIPY rotor and its derivatives has been employed successfully to measure viscosity in model lipid membranes,^16^ in protocells,^17^ and in the inner membranes of living cells.^18^ One of the main advantages of the BODIPY rotor over other reported molecular rotors is the wide dynamic range of its fluorescence response,^14^ corresponding to a broad range of viscosities, and a very weak sensitivity to solvent polarity^19^ and temperature.^16^ Control of the rotation of a fluorescent molecular rotor, imposed by mechanical bonds within the nanoconfinement^20^ provided by a MIM, however, has not been explored so far to the best of our knowledge.

Herein, we designed (Scheme 1b) and synthesized a novel electrochemically switchable bistable [2]rotaxane (**6**^4+^) with an embedded fluorescent rotor where the fluorescence output is actuated by an electrochemical stimulus. The bistable [2]rotaxane (**6**^4+^) is comprised of the π-electron-poor cyclobis(paraquat-*p*-phenylene) CBPQT^4+^ ring and a dumbbell containing a π-electron-rich tetrathiafulvalene (TTF) unit with a fluorescent BODIPY rotor acting as a stopper. This particular bistable [2]rotaxane has only one switchable recognition unit and therefore exists as a single translational coconformation in its ground state. Upon complete oxidation of the TTF unit to the stable, doubly charged TTF^2+^ dication, the CBPQT^4+^ ring is forced into juxtaposition with the BODIPY rotor as a result of Coulombic repulsions. The nature of the switching process has been investigated using (i) 1D and 2D NMR spectroscopies, (ii) steady-state and ultrafast time-resolved spectroscopies, (iii) electrochemical experiments, and (iv) quantum mechanical calculations. In low-polarity solvents, e.g., PhMe, oxidation of the TTF unit to a TTF^2+^ dication drives the CBPQT^4+^ ring toward the BODIPY rotor, increasing the fluorescence quantum yield of the latter. This remarkable fluorescence enhancement is a consequence of the increased energy barrier associated with its excited-state rotation which is responsible for its high nonradiative rate constant. The novelty of our approach lies in the ability to control the rotation of a molecular rotor by the constrained nanoconfined^20^ environment introduced by the mechanical bonding associated with this electrochemically switchable bistable [2]rotaxane (Scheme 1b). The bistable [2]rotaxane can therefore be considered as a model electromechanical *molecular brake* based on Coulombic repulsion and actuated by redox inputs, where the coconformational change is readily monitored through the fluorescence output. On the other hand, in high-polarity solvents, e.g., MeCN, an excited charge-shifted (CS) state involving electron transfer from the BODIPY rotor to the CBPQT^4+^ ring becomes energetically accessible, enforcing association between the two components and hence decreasing the fluorescence output of the BODIPY rotor. The ultrafast, photoinduced electron transfer from the BODIPY rotor to the CBPQT^4+^ ring in MeCN is corroborated by femtosecond transient absorption (fsTA) spectroscopy, which reveals the characteristic absorption features of the reduced CBPQT^4+^ ring. The unconventional electrochromic behavior of the fluorescent molecular rotor embedded within this bistable [2]rotaxane, generated by the mechanical motion of the redox-actuated switching process, demonstrates that mechanical bonding remains a powerful strategy to create unpredictable emergent properties in MIMs through the induced nanoconfinement effect. This electrochemically addressable fluorescent bistable rotaxane is of particular interest for the development of electro-optical applications because, in contrast with conventional electro-optical molecular switches, the change in fluorescence output in the rotaxane-based system is modulated by mechanical movement within the MIM, which can lead to more sophisticated outcomes.

## Results and Discussion

### Synthesis and NMR Spectroscopy

The 1,3-dipolar cycloadditions^21^ of azides with alkynes, otherwise known as the copper-catalyzed azide–alkyne cycloaddition (CuAAC) approach to synthesis, has been demonstrated^22^ to be highly effective when employed in the final step, leading to formation of mechanical bonds in the syntheses of MIMs, such as rotaxanes. The CuAAC reaction occurs readily under very mild conditions and at room temperature, a quality that is desirable for the template-directed synthesis of MIMs on account of optimal stabilization of the supramolecular intermediates.

TTF and its derivatives have been used widely in the design and synthesis of reversible, electrochemically switchable, supramolecular systems^23^ and MIMs.^24,25^ The π-electron-rich neutral TTF can be oxidized in two steps at mild potentials to give a stable radical cation and dication, respectively. In its neutral state, the TTF unit is bound strongly^23a^ inside the π-electron-poor CBPQT^4+^. Oxidation, however, leads to ejection of the oxidized TTF^2+^ dication from inside the tetracationic cyclophane as a consequence of Coulombic repulsions.

**Scheme 2:**
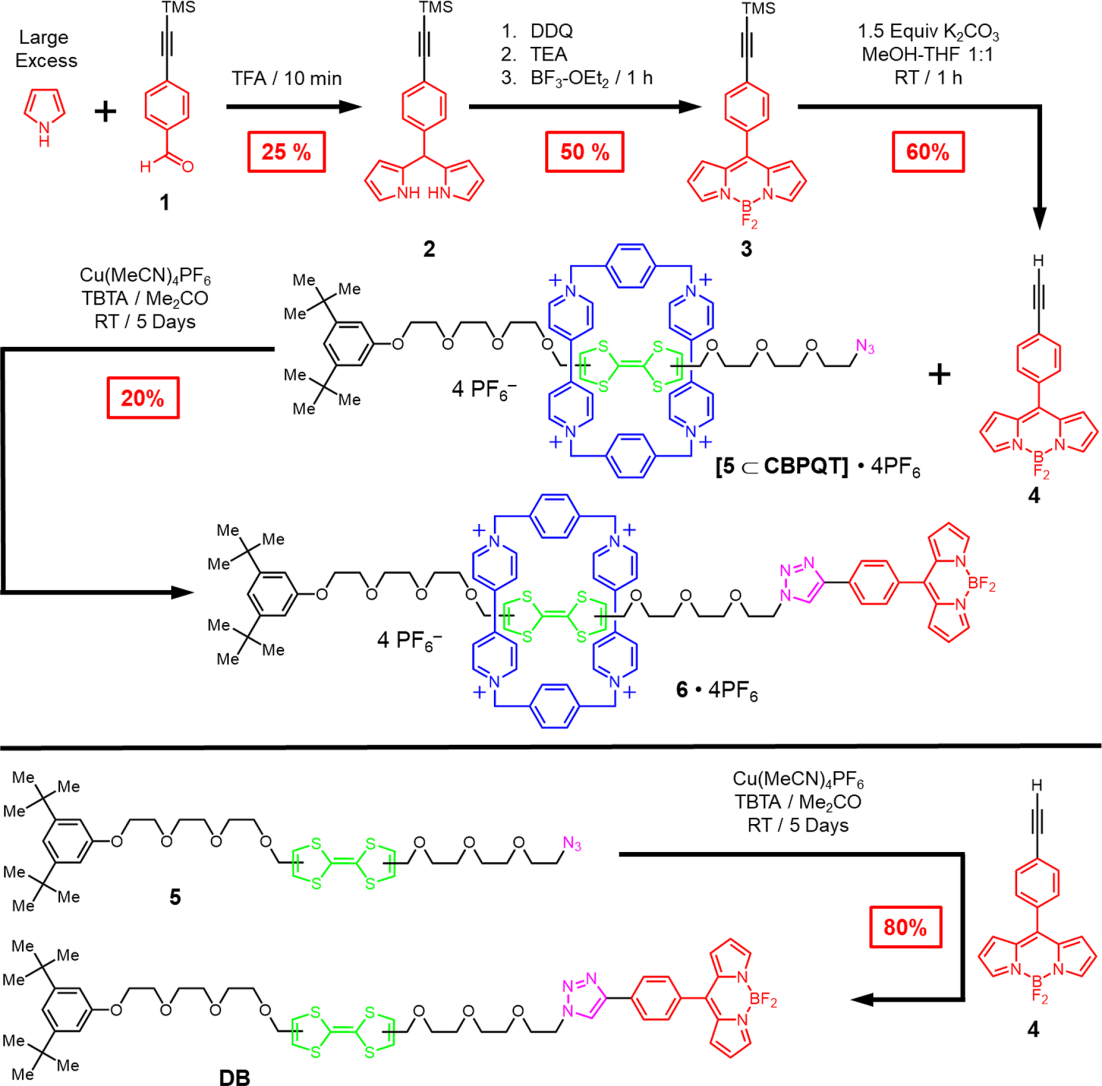
Synthesis of the BODIPY-alkyne **4**, the Bistable [2]Rotaxane **6**·4PF_6_, and the Dumbbell **DB**

The BODIPY-alkyne **4**, which was synthesized following a method reported in the literature by Wagner and Lindsey,^26^ was obtained (Scheme 2) in four steps. As for the synthesis of the *meso*-substituted dipyrromethanes **2**, we adopted a one-flask synthesis, which was developed previously for preparation of trans-substituted porphyrins.^27^ Pyrroles readily undergo acid-catalyzed condensation at room temperature in the presence of highly electrophilic carbonyl compounds, such as aldehyde **1**, which is used to form the *meso* bridge.^28^ In order to avoid oligomerization, a large excess of pyrrole is employed. Moreover, pyrrole serves as the solvent for the reaction, leading to direct formation of dipyrromethane **2**.

The π-electron-rich TTF derivative **5** was prepared using a previously reported procedure.^29^ Upon mixing of **5** with 1 mol equiv of CBPQT·4PF_6_ in Me_2_CO, the solution immediately turned an intense emerald green color, indicating formation of a donor–acceptor pseudorotaxane complex, namely, [**5**⊂CBPQT]·4PF_6_. Reaction with tetrakis(acetonitrile)copper(I) hexafluorophosphate in the presence of tris[(1-benzyl-1*H*-1,2,3-triazol-4-yl)methyl]amine (TBTA) at 20 °C for 5 days promoted the desired click reaction, affording the bistable [2]rotaxane **6**·4PF_6_ as a dark green solid in 20% yield. Reference dumbbell compound **DB** was obtained (Scheme 2) under similar reaction conditions in the absence of **CBPQT**·4PF_6_. The molecular structures and compound purities were ascertained by mass spectrometry and HPLC as well as by 1D and 2D NMR spectroscopies. See the Supporting Information (SI) for detailed synthetic procedures and characterization.

**Figure 1:**
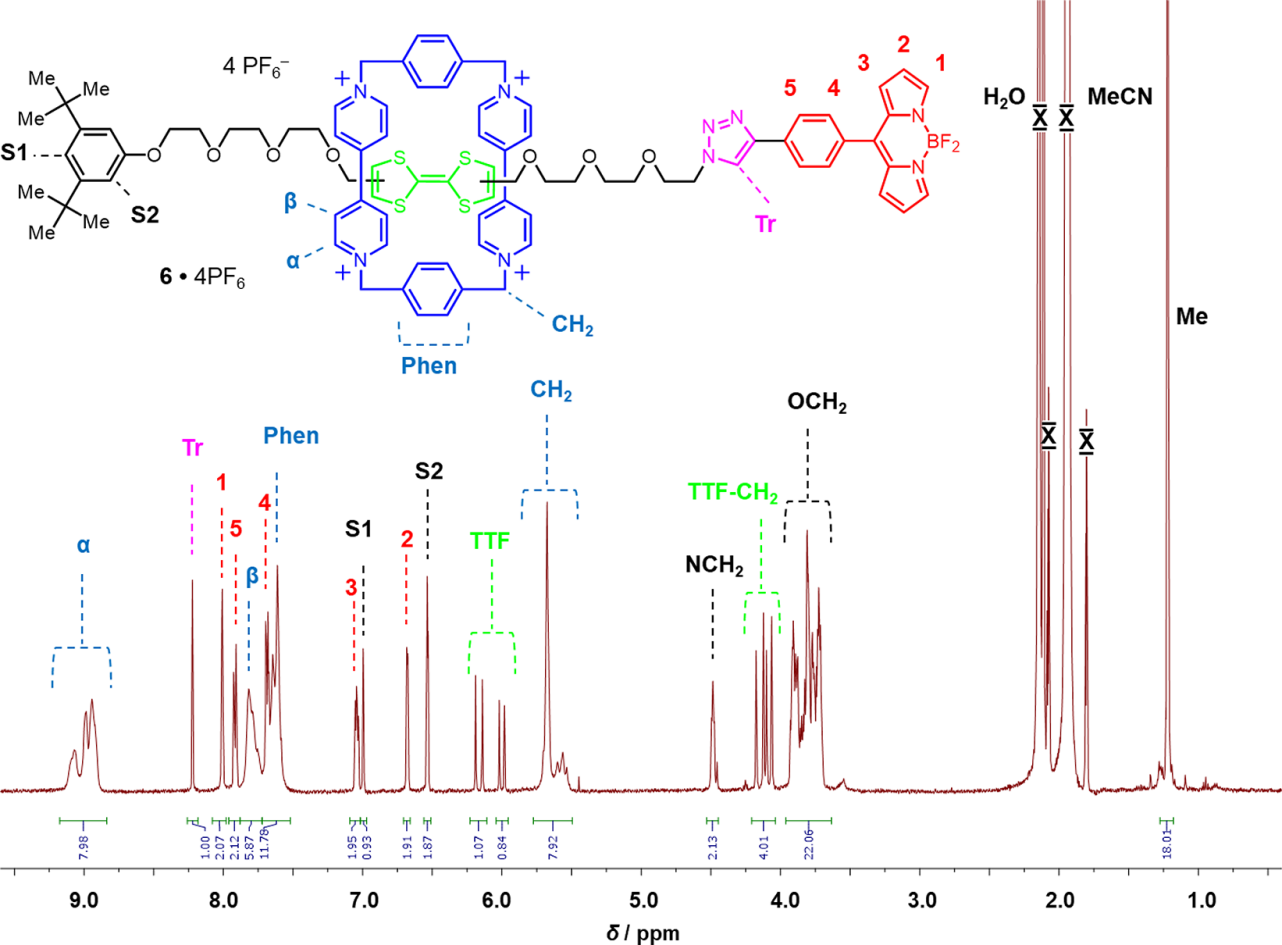
^1^H NMR spectrum (600 MHz, CD_3_CN, 298 K) of bistable [2]rotaxane **6**·4PF_6_.

The ^1^H NMR spectrum (Figure 1) of **6**·4PF_6_ confirms its mechanically interlocked nature and the fact that the CBPQT^4+^ ring encircles the TTF unit in the ground state. Notably, the separation of the signals for both the α- and the β-bipyridinium protons on the CBPQT^4+^ ring is observed at room temperature. This observation is to be expected, as the constitutional asymmetry of the dumbbell component imposes its lack of symmetry on the CBPQT^4+^ ring, rendering the α- and β-bipyridinium protons heterotopic and thus, in each case, giving rise to separate signals. We were not able to observe (Figure S8, Supporting Information) any ground-state shuttling by dynamic ^1^H NMR spectroscopy within the temperature range from 238 to 308 K, indicating that the CBPQT^4+^ ring resides, to all intents and purposes, solely on the TTF unit, at least within the limits of detection provided by variable-temperature ^1^H NMR spectroscopy. This observation is consistent with the one-station nature of the bistable [2]rotaxane when the TTF unit is neutral.

**Figure 2:**
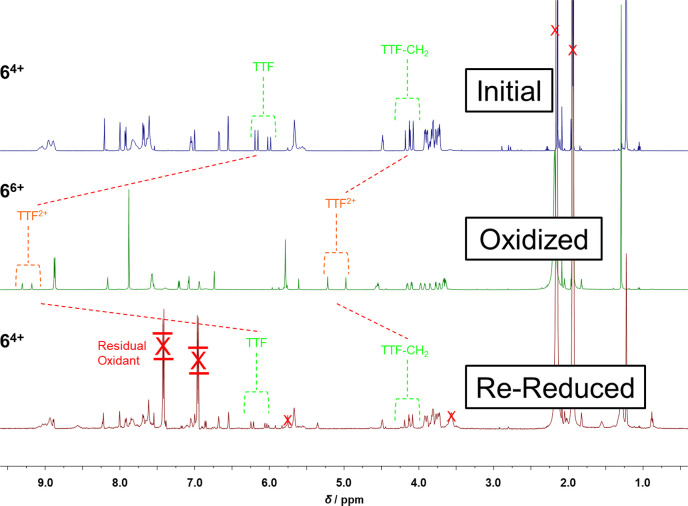
^1^H NMR spectra (600 MHz, CD_3_CN, 298 K) of the bistable [2]rotaxane **6**^4+^, fully oxidized **6**^6+^ after addition of 2 equiv of the chemical oxidant tris(4-bromophenyl)ammoniumyl hexachloroantimonate, and rereduced **6**^4+^ after addition of Zn dust. Resonances due to the chemical oxidant and residual solvent are marked with red (X) symbols.

The bistable [2]rotaxane **6**·4PF_6_ can be actuated (Scheme 1) by chemical oxidation of **6**^4+^ to **6**^6+^ in CD_3_CN using 2 equiv of tris(4-bromophenyl)ammoniumyl hexachloroantimonate as an oxidizing agent.^30^ The effects of chemical switching can be monitored by ^1^H NMR spectroscopy, steady-state UV–vis absorption, and emission spectroscopy. The ^1^H NMR spectrum of **6**^6+^ (Figure 2) is characterized by a substantial change as a consequence of large downfield shifts of the TTF^2+^ proton resonances as well as those associated with the neighboring methylene groups. These changes are in agreement with previously reported^31^ spectroscopic data. Before oxidation, the spectrum of bistable rotaxane **6**^4+^ reveals two pairs of peaks of almost equal integration (56:44), at δ = 6.19 and 6.14 ppm and δ = 6.02 and 5.98 ppm, which can be assigned to the constitutionally heterotopic TTF methine protons in the cis and trans isomers, respectively. These isomers are in dynamic equilibrium on the laboratory time scale. Following oxidation, the signals for the constitutionally heterotopic methine protons on the TTF^2+^ dication resonate^32^ at δ = 9.30 and 9.17 ppm, while those associated with the methylene protons on the adjacent CH_2_ groups are shifted downfield to 5.21 and 4.96 ppm. In this oxidized state, cis–trans isomerism is removed since the TTF^2+^ dication is no longer planar.^30a^ The movement of the CBPQT^4+^ ring from the TTF unit to the BODIPY rotor was probed (Figures S9 and S10) by two-dimensional (2D) nuclear Overhauser effect (NOE) measurements, where the presence of through-space correlations between the resonances for the BODIPY protons and those on the CBPQT^4+^ ring in **6**^6+^ were observed. It should be noted that the interaction between the CBPQT^4+^ ring and the BODIPY rotor was absent in the reduced **6**^4+^ state. The disappearance of NOE correlations between the methylene protons next to the TTF unit and those of the CBPQT^4+^ ring corroborates the switching to **6**^6+^. Upon treatment with Zn dust, TTF^2+^ was reduced (Figure 2) to its neutral form and the CBPQT^4+^ ring moved back to reside on the π-electron-rich TTF unit, as indicated by the ^1^H NMR spectrum of the product, in order to re-establish the CT interactions between TTF and the CBPQT^4+^ ring.

### Steady-State UV–vis Spectroscopy

**Figure 3:**
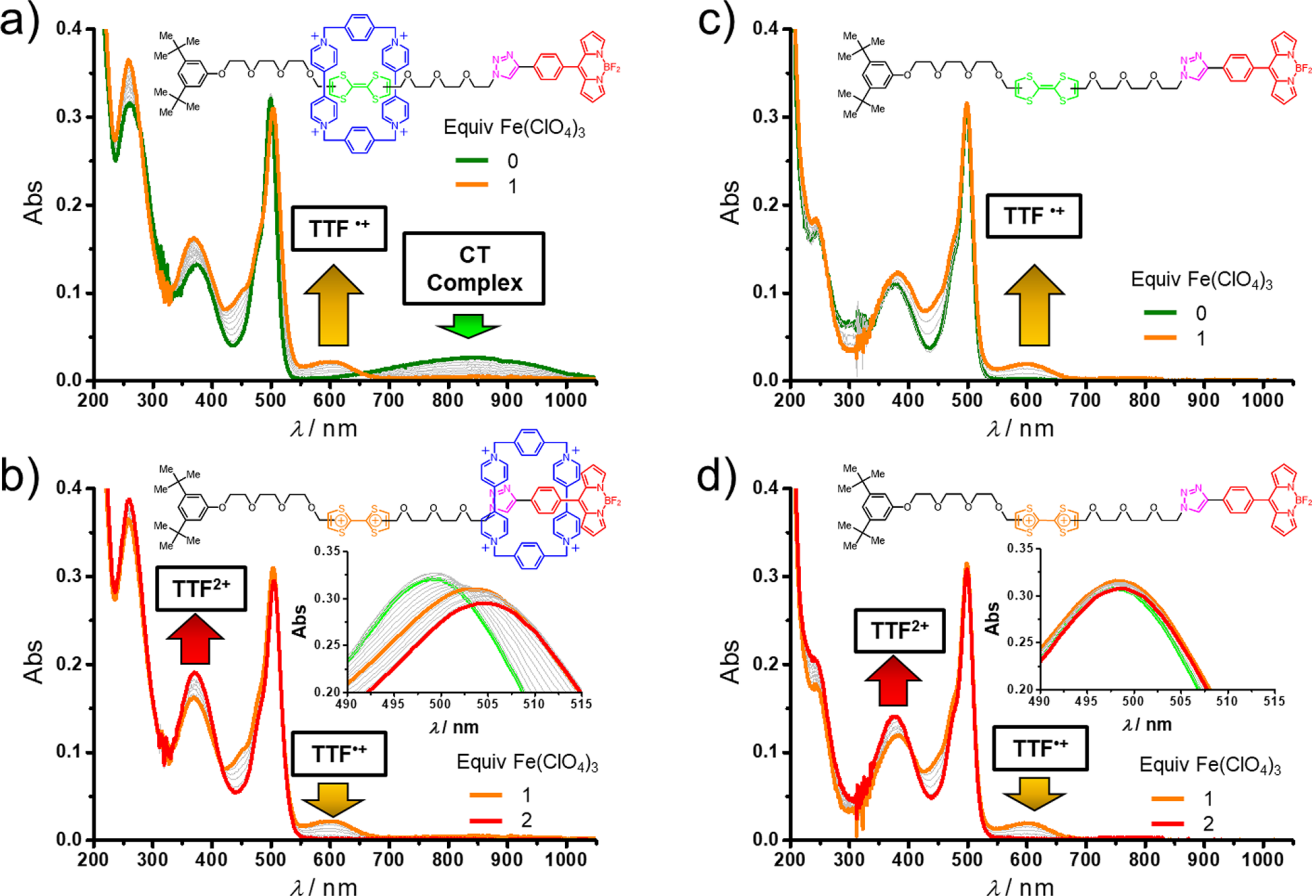
UV–vis absorption spectra of (a and b) the bistable [2]rotaxane **6**·4PF_6_ and (c and d) the reference dumbbell **DB** in MeCN (6.25 μM, 10 mm optical pathway) following addition of Fe(ClO_4_)_3_ as the chemical oxidant.

The UV–vis absorption spectrum (Figure 3a, green trace) of the bistable [2]rotaxane **6**·4PF_6_ shows the characteristic charge-transfer (CT) absorption band^33^ centered on 843 nm (ε = 3500 M^–1^ cm^–1^) for the TTF unit residing inside the CBPQT^4+^ ring. Furthermore, in the visible region, the strong absorption band at 499 nm (ε = 48 000 M^–1^ cm^–1^), typical of an S_1_ ← S_0_ electronic transition of the BODIPY chromophore unit, is observed. The UV region is characterized by the S_2_ ← S_0_ transition of BODIPY (376 nm, ε = 16 000 M^–1^ cm^–1^) and the CBPQT^4+^ absorption at 260 nm (ε = 40 000 M^–1^ cm^–1^). Switching of **6**^4+^ was investigated in a 6.25 μM solution of the 4PF_6_^–^ salt in MeCN when Fe(ClO_4_)_3_ was added.^34^ Addition of 1 mol equiv of this chemical oxidant led (Figure 3a, orange trace) to disappearance of the CT absorption band at 843 nm and the rise of absorption bands centered on 450 and 600 nm characteristic^35^ of the mono-oxidized form of the TTF. Further addition of the oxidant (up to 2 mol equiv) led to disappearance of the absorption bands for the mono-oxidized TTF unit and enhancement of the band at 375 nm (Figure 3b, red trace), indicative^35^ of TTF^2+^ dication formation. Notably, movement of the CBPQT^4+^ ring away from the TTF unit is also accompanied by a small red shift of the absorption maximum of BODIPY from 499 to 505 nm. This observed ground-state electronic perturbation is most likely caused by the enforced encirclement of the BODIPY rotor by the tetracationic cyclophane, as reference **DB** does not show (Figure 3c and 3d) any red shift upon oxidation of the TTF unit. After reduction with Zn powder, the original spectrum is quantitatively restored. In summary, both the ^1^H NMR spectroscopic and the UV–vis spectrophotometric experiments showed clearly that redox switching of the TTF unit forces the CBPQT^4+^ ring toward and away from the BODIPY rotor.

### Electrochemistry

**Figure 4:**
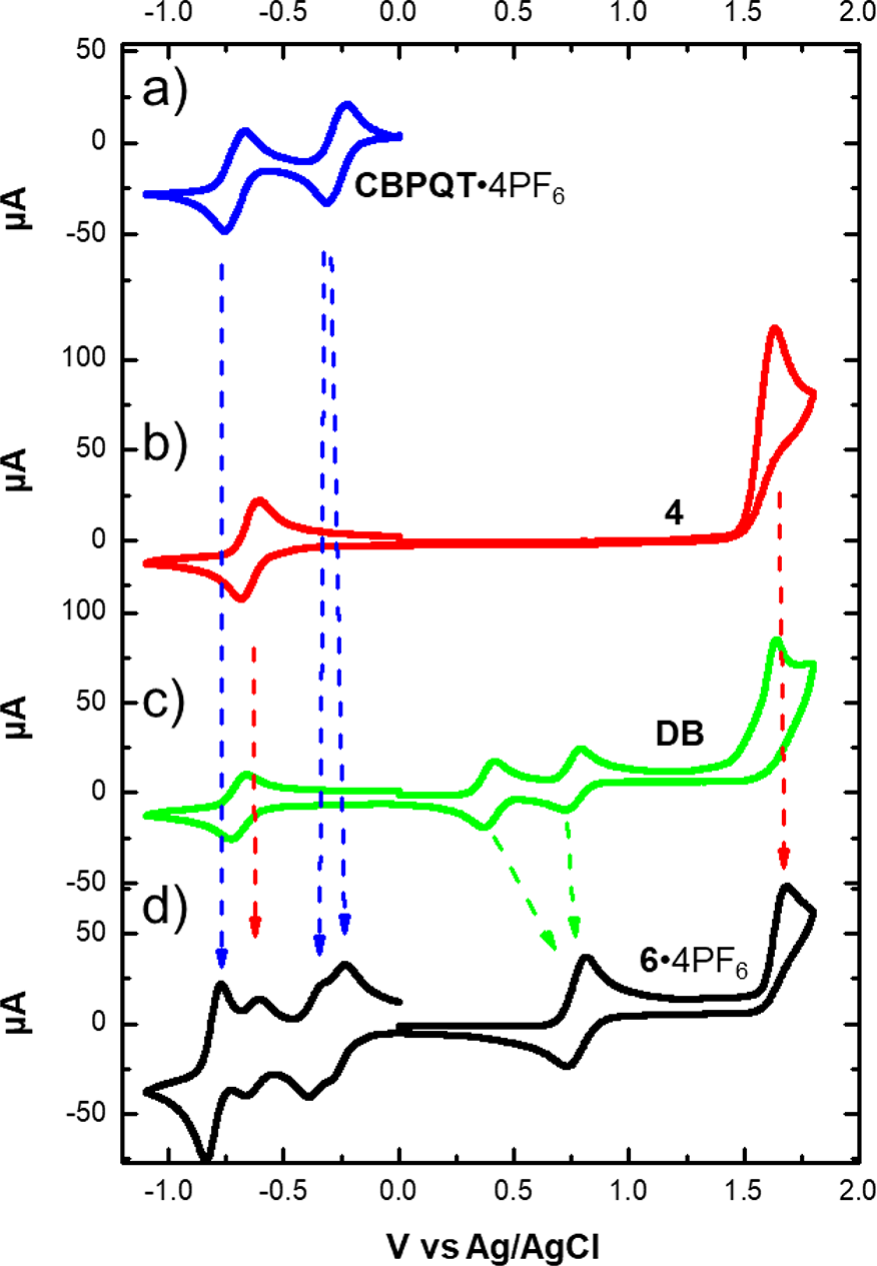
Cyclic voltammograms of (a) the ring component **CBPQT**·4PF_6_, (b) the BODIPY-alkyne **4**, (c) the dumbbell **DB**, and (d) the bistable [2]rotaxane **6**·4PF_6_.

Switching of the bistable [2]rotaxane can also be enacted electrochemically. The CBPQT^4+^ ring shows (Figure 4a) two characteristic reversible two-electron reductions with *E*_1/2_ at −270 and −715 mV vs Ag/AgCl, while the BODIPY rotor reveals (Figure 4b) a reversible one-electron reduction at *E*_1/2_ = −652 mV and an irreversible oxidation peak at +1630 mV. The TTF oxidation processes, which are TTF → TTF^•+^ followed by TTF^•+^ → TTF^2+^ one-electron events, are well resolved in the CV of **DB** (Figure 4c) with *E*_1/2_ at +392 and +755 mV, respectively. On the other hand, the oxidative scan of the bistable [2]rotaxane **6**·4PF_6_ (Figure 4d) shows only one peak centered at +772 mV encompassing both of the one-electron processes, which together generate the TTF^2+^ dicationic state from its neutral form to the radical TTF^•+^ intermediate. Complete disappearance of the first oxidation peak matches the fact that the TTF unit within the bistable [2]rotaxane **6**·4PF_6_ is encircled completely by the electron-poor CBPQT^4+^ ring, such that the first TTF oxidation is shifted substantially to a more positive potential, close to the potential for the second oxidation.^13^ We also performed variable scan-rate CV measurements in order to elucidate the kinetics associated with the return of the CBPQT^4+^ ring from the oxidation-induced coconformation to the ground-state coconformation (GSCC). As observed in other bistable MIMs,^24e^ the initial oxidation of the TTF unit forces the CBPQT^4+^ ring onto the alternative recognition site, and the subsequent rereduction of the TTF unit back to its neutral form does not result immediately in the regeneration of the GSCC. The transient coconformation, where the CBPQT^4+^ ring still encircles the weaker binding site while the TTF unit is back in the neutral state, is referred to as the metastable-state coconformation (MSCC). As the conversion of the MSCC to the GSCC is usually an activated process, its kinetics have a measurable rate, from seconds to hours, which depends on the nature of the linker, the temperature, and the environment.^31^ In the case of the bistable [2]rotaxane **6**^4+^, however, even at a high scan rate (2000 mV/s, Figure S11), we could not detect the presence of any MSCC, which would appear as emergence of the first oxidation of free TTF in the second scan. The absence of any detectable MSCC suggests that there is not much, if any, affinity between the CBPQT^4+^ ring and the BODIPY rotor, and their association after the oxidation step is driven largely by the Coulombic repulsion between the tetracationic cyclophane and the TTF^2+^ dication.

### Quantum Mechanical Calculations

**Figure 5:**
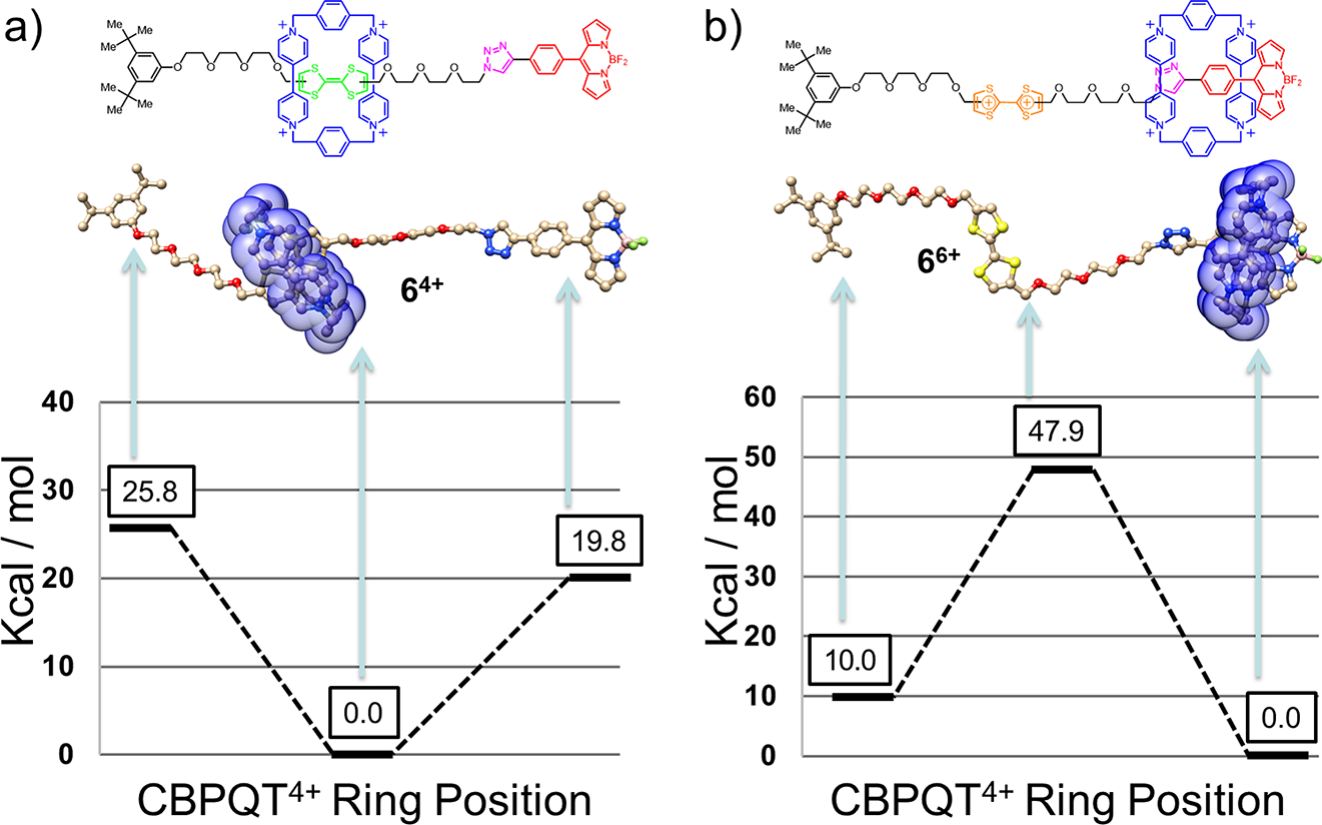
DFT-calculated relative energy surface diagrams of (a) **6**^4+^ and (b) **6**^6+^. Compound **6**^4**+**^ exists as a single ground-state coconformation. Oxidizing the TTF-containing dumbbell unit to afford **6**^6**+**^ dramatically increases the Columbic repulsion of TTF^2+^ with the CBPQT^4+^ ring, causing it to translate wholly to the site located on the BODIPY unit.

In order to examine the energetics that govern the intramolecular noncovalent bonding interactions in the bistable [2]rotaxane **6**·4PF_6_ in its different redox states, we performed density functional theory (DFT) calculations at the M06-2X/6-311++G**//M06-2X/6-31G* level that includes corrections for van der Waals attraction (normally not included in DFT).^36^ Although **6**^4+^ has multiple rotatable C–O and C–C single bonds, we started with the linear conformation that has the longest possible distance between the TTF at the center and the two ends of **6**^4+^. This conformation minimizes the electrostatic repulsion between TTF^2+^ and CBPQT^4+^ once TTF is oxidized and CBPQT^4+^ is driven to one of the two ends. We assume that the entropic contribution to the free energy from the multiple rotatable single bonds will cancel out as CBPQT^4+^ relocates. The ground-state geometries and energy landscapes predicted from the DFT methods (Figure 5), carried out on the **6**^4+^ and **6**^6+^ states, are in agreement with the experimental observations. In **6**^4+^ the CBPQT^4+^ ring prefers to reside on the TTF unit rather than on the BODIPY rotor by 19.8 kcal/mol due to stronger donor–acceptor interactions between CBPQT^4+^ and TTF. Once two electrons are removed from the TTF unit, the strong electrostatic repulsion between TTF^2+^ and CBPQT^4+^ drives the ring to relocate preferentially beside the BODIPY rotor, leading to a 47.9 kcal/mol energy stabilization. The other coconformation of **6**^6+^ has the CBPQT^4+^ ring move to the di-*tert*-butyl benzene unit, which is 10.0 kcal/mol higher in energy than when the CBPQT^4+^ ring resides beside the BODIPY rotor.

### Redox Modulation of Excited-State Properties

**Figure 6:**
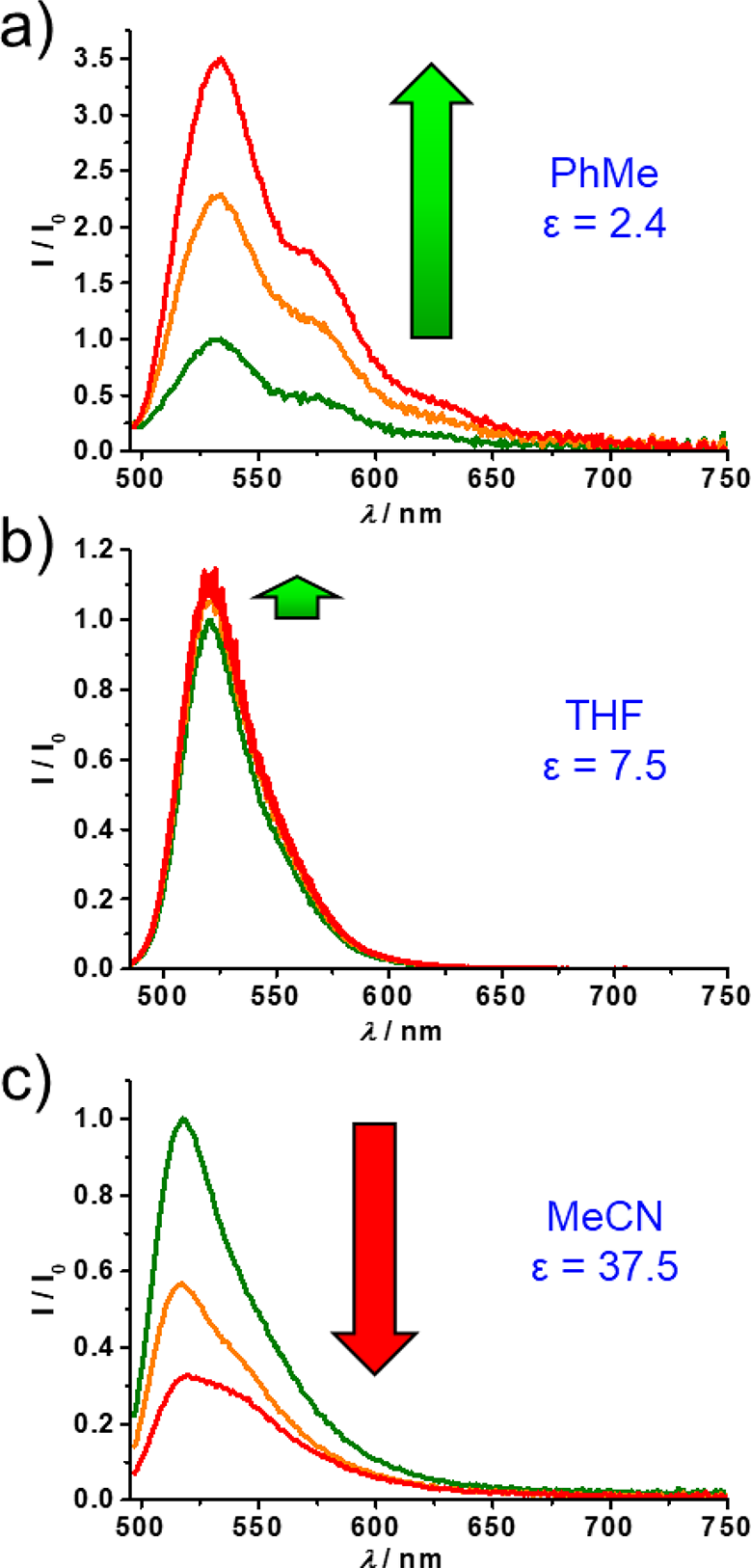
Fluorescence spectral changes of the bistable [2]rotaxane **6**·4PF_6_ (green traces) in (a) PhMe, (b) THF, and (c) MeCN following addition of 1 equiv (orange traces) and 2 equiv (red traces) of Fe(ClO_4_)_3_ as the chemical oxidant.

In order to demonstrate the ability of the bistable [2]rotaxane to modulate fluorescence outputs and excited-state dynamics of the BODIPY rotor, we performed steady-state fluorescence titration and fsTA measurements before and after chemical oxidation. A dilute solution of **6**·4PF_6_ in PhMe excited at 467 nm shows (Figure 6a, green trace) the characteristic emission maximum of BODIPY around 520 nm. The measured fluorescence quantum yield is low (Φ_em_ = 0.19%) and insensitive to the solvent polarity, as observed (Figure 6b and 6c, green traces) in both MeCN and THF. These results are consistent^37^ with those reported for sterically unhindered BODIPY derivatives. Upon addition of 2 mol equiv of Fe(ClO_4_)_3_, a 3.4-fold increase in fluorescence intensity was observed for **6**^6+^ in PhMe. For comparison, the fluorescence signal of the BODIPY unit in the reference dumbbell **DB**, measured (Figure S12) before and after oxidation of the TTF unit, does not show an appreciable change in the tested solvents. We attribute the fluorescence signal enhancement of the oxidized bistable [2]rotaxane to the expected increase in the energy barrier for intramolecular rotation of the BODIPY rotor induced by the enforced positioning of the CBPQT^4+^ ring close to the rotor in the **6**^6+^ state. Unexpectedly, when the same fluorescence titration experiment of the bistable [2]rotaxane is performed in MeCN (Figure 6c), a nearly 3-fold decrease in the fluorescence quantum yield is observed. In THF (Figure 6b), which has an intermediate polarity compared to PhMe and MeCN, neither an increase nor a decrease in the fluorescence signal is observed upon addition of Fe(ClO_4_)_3_. The different fluorescence responses of [2]rotaxane **6**·4PF_6_, upon chemical switching in the three solvents, suggests that an additional excited-state reaction, which is highly sensitive to the solvent polarity, must be involved. We hypothesize that the quenching of fluorescence in MeCN could be indicative of a photoinduced electron-transfer reaction from the BODIPY singlet excited state to the electron-accepting CBPQT^4+^ ring. In order to obtain support for this hypothesis, we performed fsTA spectroscopic investigations.

**Figure 7:**
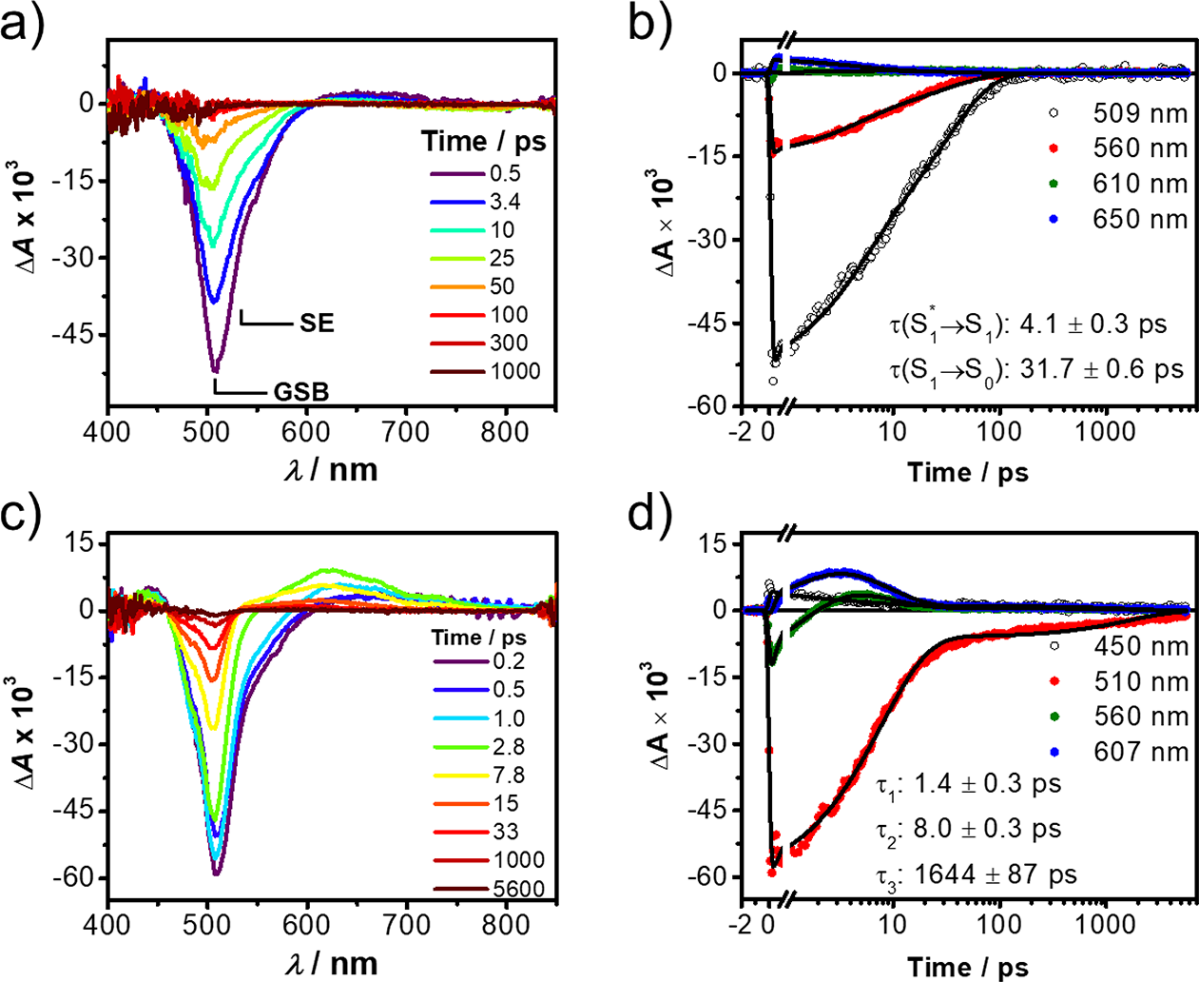
fsTA spectroscopy of the bistable [2]rotaxane **6**·4PF_6_ in MeCN (a and b) before and (c and d) after addition of 2 equiv of Fe(ClO_4_)_3_ as the chemical oxidant. Samples were excited at 497 nm with a ∼150 fs laser pulse. (a and c) fsTA spectra at selected delay times; (b and d) kinetic fits at selected wavelengths.

**Figure 8:**
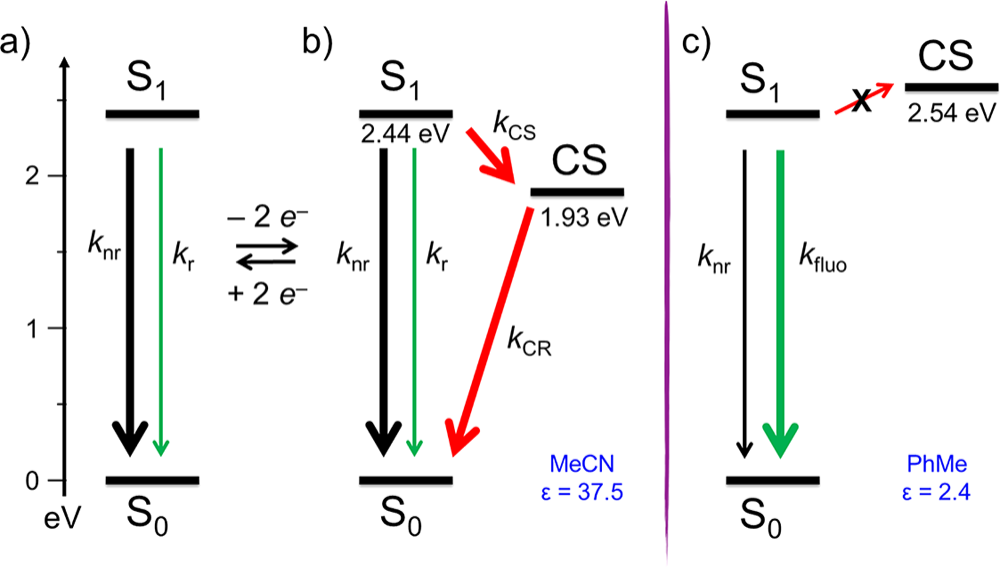
Energy diagram showing excited-state decay pathways of the bistable [2]rotaxane **6·**4PF_6_ in MeCN (a) before and (b) after the addition of 2 equiv of Fe(ClO_4_)_3_ as the chemical oxidant. In a low-polarity solvent such as PhMe (c), the photoinduced electron-transfer reaction is energetically unfavorable and the fluorescence decay is enhanced on account of the hindered intramolecular binding rotation responsible for the nonradiative decay process.

Photoexcitation of the bistable [2]rotaxane **6**·4PF_6_ in MeCN at 497 nm with a 150 fs laser pulse populated the lowest excited singlet state of the BODIPY rotor, which displays a prominent ground-state bleach (GSB) at 500 nm and overlaps partially with stimulated emission (SE) signals in the 500–600 nm region (Figure 7a). These transient features decay biexponentially with time constants of τ _S1*→S1_ = 4.1 ± 0.3 ps and τ_S1→S0_ = 31.7 ± 0.6 ps. The first decay component can be assigned to the solvation evolution or vibrational/conformational relaxation associated with the BODIPY core in the excited state, while the relaxed excited singlet state decays with the slower time constant to the ground state. On the other hand, photoexcitation of the oxidized [2]rotaxane **6**^6+^ under the same conditions generates ^1^*BODIPY, which decays rapidly (τ_CS_ = 1.4 ± 0.3 ps) to produce (Figure 7c) a new species with an absorption maximum at 610 nm that is indicative^38^ of the presence of the CBPQT^3+•^ (Figure S14). The free-energy change for such a process in polar MeCN can be estimated as Δ*G*_CS_ = *e*(*E*_OX_ – *E*_RED_) – *E*_S_, where *E*_OX_ is the oxidation potential (+1.63 V, Figure 4) of the BODIPY rotor and *E*_RED_ is the reduction potential (−0.27 V, Figure 4) of the CBPQT^4+^, while *E*_S_ is the energy of ^1^*BODIPY (+2.44 eV, from the average of absorption and fluorescence maxima), so that Δ*G*_CS_ = −0.54 eV, and is thus consistent with the observed fluorescence-quenching results. The Δ*G*_IP_ for formation of an ion pair in a solvent of arbitrary polarity can be determined using an expression developed by Weller:^39^
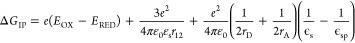
1where the redox potentials are measured in a high-polarity solvent with a static dielectric constant ε_sp_, *e* is the charge of the electron, *r*_12_ is the ion-pair distance, *r*_D_ and *r*_A_ are the ionic radii, and ε_s_ is the static dielectric constant of a solvent of arbitrary polarity. While the term involving the ion-pair distance is the Coulombic interaction of the ions, the last term accounts for the lesser ability of the lower-polarity solvents to stabilize charges as compared to the higher polarity used in the electrochemical measurements. There are several limitations, however, with this treatment for estimating Δ*G*_IP_ for large π-stacked donor–acceptor systems. First, the ionic radii of the donor and acceptor are larger than the distance between them, which violates the assumption of a hard-sphere model surrounded by a continuous dielectric that is intrinsic to eq 1. Second, the BODIPY unit is highly shielded from the surrounding medium by the presence of the CBPQT^4+^ ring; thus, BODIPY experiences a significant electrostatic influence from the CBPQT^4+^ ring, which will deviate from that of the bulk solvent. Nevertheless, one can estimate that the energy of the CS state is destabilized by at least 0.6 eV in PhMe relative to its energy in MeCN, making the photoinduced electron-transfer reaction endoenergetic by ≥0.1 eV.^40^ Notably, the charge recombination proceeds biexponentially. The shorter lifetime (8.0 ± 0.3 ps) can be safely assigned to the geminate recombination from TTF–CBPQT^3+•^–BODIPY^+•^ to the ground state. A smaller fraction of the population, however, can undergo to a charge-shift reaction to generate TTF^+•^–CBPQT^4+^–BODIPY^+•^, which ultimately decays in 1644 ± 87 ps. The energy levels and excited-state decay pathways under various conditions are summarized in Figure 8.

## Conclusions

We have reported the synthesis of an electrochemically switchable bistable [2]rotaxane with an embedded fluorescent molecular rotor, the BODIPY rotor, and demonstrated that the redox actuation of this mechanically interlocked molecule can impose a nanoconfined constrained environment that controls the rotation of the fluorescent rotor, resulting in a unique electrochromic effect. The electrochemically switchable [2]rotaxane, **6**·4PF_6_, composed of a CBPQT^4+^ ring mechanically interlocked with a dumbbell component containing a TTF recognition site and a functional fluorescent molecular rotor in the form of a BODIPY stopper, was synthesized in good yield by a template-directed protocol utilizing a “threading-followed-by-stoppering” approach in combination with the CuAAC reaction. The bistable [2]rotaxane can be switched reversibly so that the CBPQT^4+^ ring is positioned next to the BODIPY rotor upon oxidation of the TTF unit to its TTF^2+^ dication. We investigated the switching of the ground-state coconformation by means of 1D and 2D NMR spectroscopies and investigated the corresponding electronic excited-state dynamics changes using a variety of steady-state and time-resolved optical spectroscopies. Remarkably, two completely different mechanisms of fluorescence coexist within this fluorescent-bistable rotaxane upon switching of its ground-state coconformation: (i) fluorescence enhancement by reducing the loss of energy from the excited states to the nonradiative molecular motions of the BODIPY rotor in low-polarity solvents and (ii) fluorescence quenching on account of the photoinduced electron-transfer reaction from the BODIPY singlet excited state to the electron-accepting CBPQT^4+^ ring in polar solvents. This electrochromic effect is generated by the constrained nanoconfined environment introduced by the mechanical bond in this bistable [2]rotaxane which imparts the forced association between the CBPQT^4+^ ring and the BODIPY rotor, driven purely by the Coulombic repulsion between the tetracationic ring and the TTF^2+^ dication on the dumbbell. We believe that the extension of the concept of the nanoconfinement effect,^20^ introduced by mechanical bonding in the context of stimuli-responsive materials based on MIMs, could lead to novel electro-optical switchable materials that can perform complex operations, holding promise for future applications as sensors, molecular memories, and molecular logic gates.

## Supporting Information Available

Experimental details, including synthesis, NMR, and supportive figures; additional electrochemical studies and fluorescence spectra; quantum mechanical calculations (PDF)
