# Supplementary material for: Electrochemical Switching of a Fluorescent Molecular Rotor Embedded within a Bistable Rotaxane
Source: J Am Chem Soc. 2020 May 29;142(27):11835–46. doi: 10.1021/jacs.0c03701 (PMC8007092; doi:10.1021/jacs.0c03701)
Supplement: Supplementary file 1 — ja0c03701_si_001.pdf [file ja0c03701_si_001.pdf]

Supporting Information

for

Electrochemical Switching of a Fluorescent Molecular Rotor Embedded within a Bistable Rotaxane

Yilei Wu,[†] Marco Frasconi,*[,§] Wei-Guang Liu,[‡] Ryan M. Young,[†] William A. Goddard III,[‡] Michael R. Wasielewski*[,†,‖] and J. Fraser Stoddart*[,†,Δ,⊥]

[†]*Department of Chemistry and* [‖]*Institute for Sustainability and Energy at Northwestern (ISEN), Northwestern University, 2145, Sheridan Road, Evanston, Illinois 60208-3113, United States*

[§]*Department of Chemical Sciences, University of Padova, Via Marzolo 1, Padova 35131, Italy*

[‡]*Materials and Process Simulation Center, California Institute of Technology, Pasadena, California 91125, United States*

[Δ] *Institute for Molecular Design and Synthesis, Tianjin University, 92 Weijin Road, Nankai District, Tianjin 300072, China*

[⊥]*School of Chemistry, University of New South Wales, Sydney, NSW 2052, Australia*

E-mail: marco.frasconi@unipd.it; m-wasielewski@northwestern.edu; stoddart@northwestern.edu

Table of Contents

Section A. Materials / General Methods / Instrumentation **S2**

Section B. Synthetic Protocols **S2**

Section C. NMR Spectroscopy **S6**

Section D. Electrochemistry **S14**

Section E. Fluorescence Spectroscopy **S15**

Section F. Femtosecond Transient Absorption (fsTA) Spectroscopy **S16**

Section G. Quantum Mechanical Calculations **S17**

Section H. References **S19**

Section A. Materials / General Methods / Instrumentation

All reagents were purchased from commercial suppliers and used without further purification unless stated otherwise. The TTF derivative **5** was prepared using a previously reported procedure.[S1] Analytical high pressure liquid chromatography (HPLC) was performed on reverse phase-HPLC (RP-HPLC) instruments, using a biphenyl stationary phase and a binary solvent system (MeCN and H_2O with 0.1% CF_3CO_2H). Thin layer chromatography (TLC) was performed on silica gel 60 F254 (E. Merck). Column chromatography was carried out on silica gel 60F (Merck 9385, 0.040–0.063 mm). High-resolution mass spectra were measured on an Agilent 6210 Time of Flight (TOF) LC-MS, using an ESI source, coupled with an Agilent 1100 HPLC stack, using direct infusion (0.6 mL/min). Nuclear magnetic resonance (NMR) spectra were recorded on a Bruker Avance 600 and 500 spectrometers, with working frequencies of 600 and 500 MHz (^{1}H), respectively, and at 150 and 125 MHz (^{13}C), respectively. Chemical shifts are reported in ppm relative to the signals corresponding to the residual non-deuterated solvents (CD_3CN: δ_H = 1.94 ppm and δ_C = 1.32 and 118.26 ppm). The temperature (T) was calibrated to the temperature-dependent chemical shift of methanol for T < 295 K and ethylene glycol for T > 295 K.

Section B. Synthetic Protocols

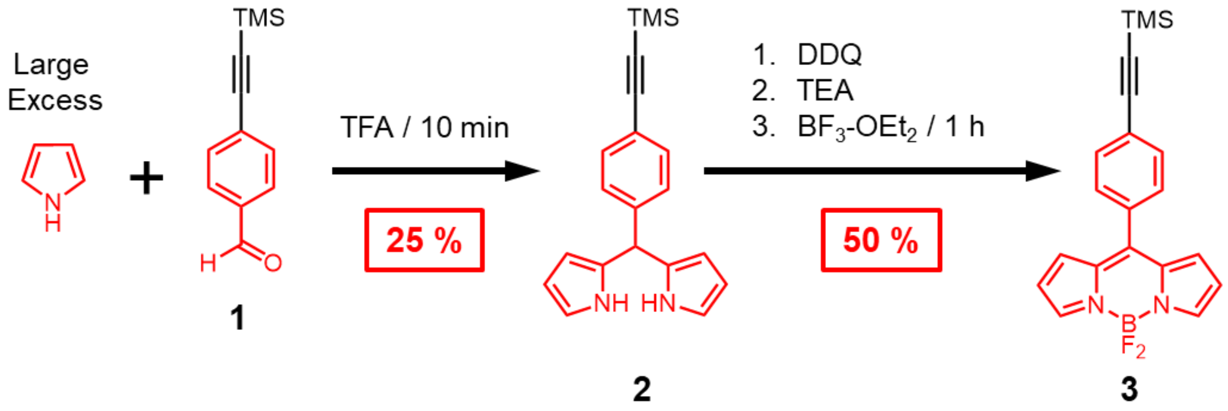

3. 4-[(Trimethylsilyl)ethynyl]benzaldehyde (5 g, 24.7 mmol) was dissolved in freshly distilled pyrrole (64.1 ml, 0.989 mol) and the mixture was degassed with Ar for 10 min. TFA (0.42 ml) was

then added and the mixture was stirred at room temp under Ar for 10 min. DDQ (5.61 g, 24.7 mmol) was then added and the mixture was stirred at room temp for 1 h. Triethylamine (24.1 ml, 0.173 mol) was then added followed by BF_3OEt_2 (21.9 ml, 0.173 mol). The solution was then stirred at room temp for 16 h. The solution was then filtered through a pad of celite and the filtrate was washed with water (4 x 75 ml), dried ($MgSO_4$), filtered and evaporated in vacuo. The residue was purified by column chromatography (SiO_2) eluting with 5:95 EtOAc:CH_2Cl_2 to yield the product (1.17 g, 13%). ^{1}H NMR (500 MHz, CD_3CN, ppm): δ_H 7.97 (t, J = 1.5 Hz, 2H), 7.61 (s, 4H), 7.01 (d, J = 4.2 Hz, 2H), 6.62 (dt, J = 4.1, 1.5 Hz, 2H), 0.27 (s, 9H). ^{13}C NMR (125 MHz, CD_3CN, ppm): δ_C 147.6, 145.4, 135.6, 134.6, 132.7, 132.7, 131.8, 126.3, 120.0, 104.8, 98.0, -0.2.

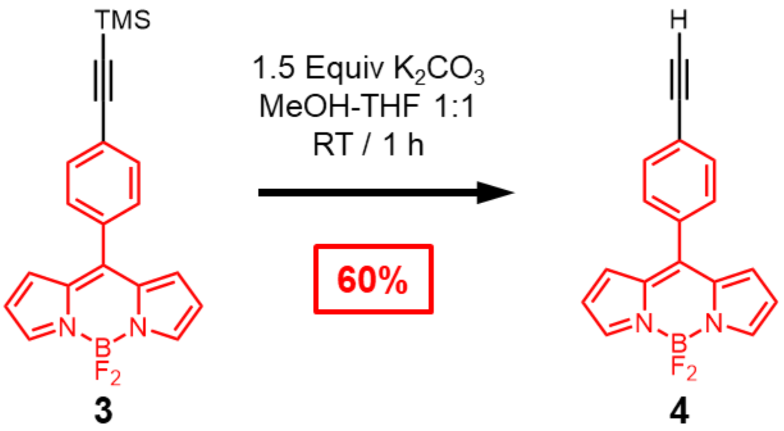

4. **3** (390 mg, 1.07 mmol) was dissolved in 1:1 MeOH:THF (20 ml) and anhydrous K_2CO_3 (216 mg, 1.51 mmol) was added and the mixture was stirred at room temp for 1 h. CH_2Cl_2 (80 ml) was then added and the mixture was washed with H_2O (3 x 50 ml), dried ($MgSO_4$) and evaporated in vacuo. The residue was purified by column chromatography (SiO_2) eluting with 3:2 hexane: CH_2Cl_2 to yield the pure product (137 mg, 66%). ^{1}H NMR (500 MHz, CD_3CN, ppm): δ_H 7.97 (s, 2H), 7.69 (d, J = 7.9 Hz, 2H), 7.63 (d, J = 8.0 Hz, 2H), 7.02 (d, J = 4.3 Hz, 2H), 6.63 (d, J = 4.6 Hz, 1H), 3.60 (s, 1H). ^{13}C NMR (125 MHz, CD_3CN, ppm): δ_C 147.5, 145.5, 135.6, 134.8, 133.0, 132.8, 125.5, 120.0, 120.0, 118.3, 83.3, 81.4.

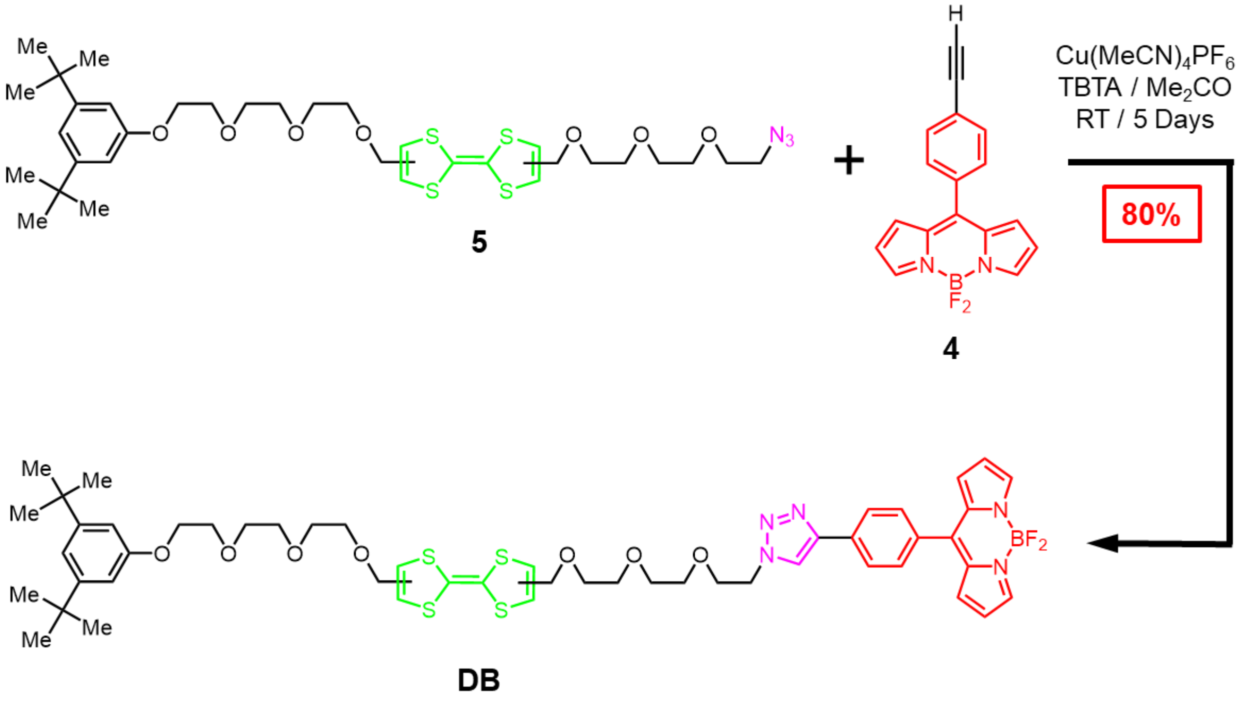

DB. A solution of **5**[S1] (25 mg, 0.034 mmol), **4** (10 mg, 0.034 mmol), TBTA (1.8 mg, 0.0034 mmol), and $Cu(MeCN)_4PF_6$ (1.3 mg, 0.0034 mmol) in anhydrous Me_2CO (7 mL) were stirred for 5 days at room temp. The solvent was then evaporated off and the resulting brown solid was purified by column chromatography (SiO_2: EtOAc as the eluent) to afford the desired product (28 mg, 80 %). ^{1}H NMR (500 MHz, CD_3CN, ppm): δ_H 8.33 (s, 1H), 8.09 – 8.01 (m, 2H), 7.96 (s, 2H), 7.72 (dd, *J* = 8.3, 2.9 Hz, 2H), 7.13 – 7.07 (m, 2H), 7.06 – 7.01 (m, 1H), 6.74 (t, *J* = 1.7 Hz, 2H), 6.63 (dd, *J* = 4.6, 1.7 Hz, 2H), 6.32 – 6.24 (m, 2H), 4.62 – 4.56 (m, 2H), 4.21 (dd, *J* = 4.2, 1.1 Hz, 2H), 4.17 (dd, *J* = 2.2, 1.1 Hz, 2H), 4.09 (dt, *J* = 7.1, 2.6 Hz, 2H), 3.94 – 3.88 (m, 2H), 3.78 – 3.72 (m, 2H), 3.64 – 3.46 (m, 16H), 1.28 (d, *J* = 1.1 Hz, 18H). ^{13}C NMR (125 MHz, CD_3CN, ppm): δ_C 156.4, 145.7, 140.8, 138.1, 131.5, 131.0, 130.6, 130.4, 129.9, 126.9, 64.5, 33.6.

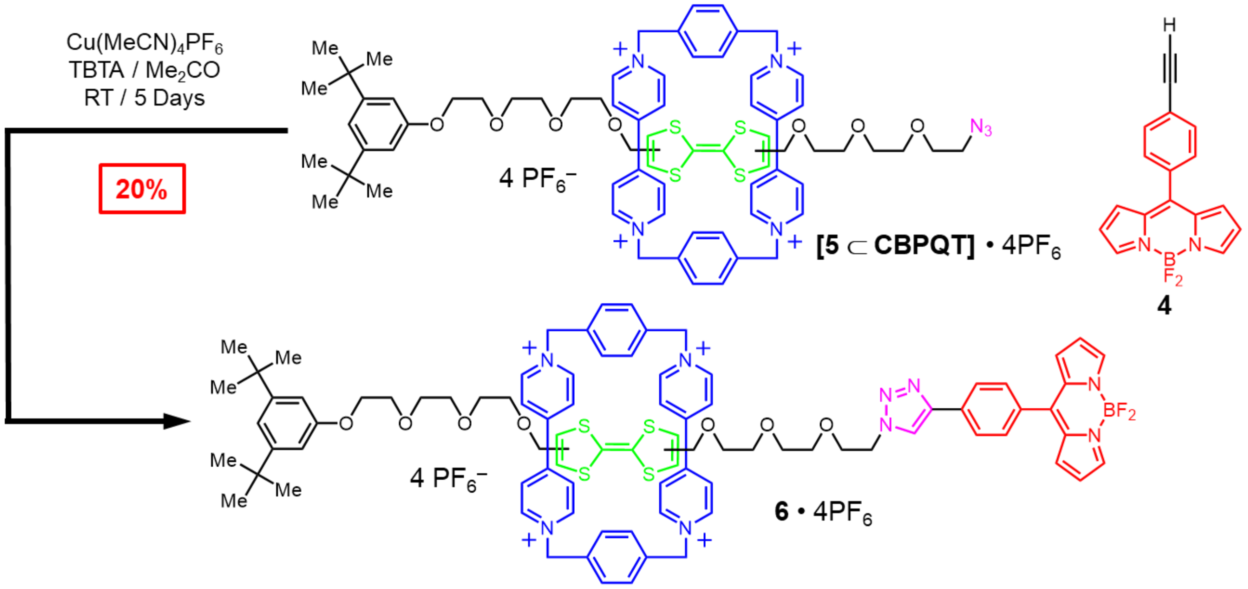

6•4PF$_6$. A solution of **5**[S1] (25 mg, 0.034 mmol), **4** (10 mg, 0.034 mmol), CBPQT.4PF$_6$ (45 mg, 0.041 mmol), TBTA (1.8 mg, 0.0034 mmol), and Cu(MeCN)$_4$PF$_6$ (1.3 mg, 0.0034 mmol) in anhydrous Me$_2$CO (7 mL) were stirred for 5 days at room temp. The solvent was then evaporated off and the resulting green solid was purified by column chromatography (SiO$_2$: 0.5 % w/v Me$_2$CO solution of NH$_4$PF$_6$ as the eluent). The green fraction in Me$_2$CO was collected, and concentrated to a minimum volume, before the crude product was precipitated by the addition of H$_2$O. The resulting solid was collected by filtration to afford the [2]rotaxane **6**·4PF$_6$ (14.5 mg, 20%) as a green powder. ^{1}H NMR (600 MHz, CD$_3$CN, 238 K, ppm): δ_H 9.05 (d, *J* = 6.3 Hz, 1H), 8.98 (d, *J* = 6.5 Hz, 1H), 8.93 (d, *J* = 6.5 Hz, 1H), 8.88 (d, *J* = 6.4 Hz, 2H), 8.85 – 8.79 (m, 4H), 8.20 (s, 1H), 8.01 (s, 2H), 7.83 – 7.80 (m, 2H), 7.75 – 7.52 (m, 20H), 7.49 (s, 1H), 7.46 (s, 1H), 7.00 (dd, *J* = 13.7, 4.1 Hz, 2H), 6.93 – 6.91 (m, 1H), 6.68 (dd, *J* = 4.2, 1.9 Hz, 2H), 6.42 (dd, *J* = 12.9, 1.6 Hz, 2H), 6.25 (s, TTF-H, 0.5H), 6.14 (s, TTF-H, 0.5H), 6.04 (s, TTF-H, 0.5H), 5.92 (s, TTF-H, 0.5H), 5.64 – 5.53 (m, 6H), 5.47 (d, *J* = 13.7 Hz, 1H), 5.41 (d, *J* = 13.5 Hz, 1H), 4.45 – 4.39 (m, N-CH$_2$, 2H), 4.16 (s, TTF-CH$_2$, 1H), 4.08 (s, TTF-CH$_2$, 1H), 4.04 (s, TTF-CH$_2$, 1H), 3.99 (s, TTF-CH$_2$, 1H), 3.90 – 3.59 (m, O-CH$_2$, 20H), 1.16 (s, -CH$_3$, 9H), 1.15 (s, CH$_3$, 9H). ^{13}C NMR (125 MHz, CD$_3$CN, 298 K, ppm): δ_C 159.2, 153.2, 147.9, 146.7, 145.5, 145.3, 145.2, 136.6, 135.5, 134.4, 133.7, 133.5, 133.4, 132.6, 132.5, 131.6, 126.5, 126.2, 126.2, 123.0, 122.9, 120.4, 120.4, 119.9, 119.1, 118.9, 116.1, 116.0, 109.6, 108.7, 108.6, 71.4, 71.3, 71.3, 71.2, 71.2, 70.8, 70.7, 70.6, 70.6, 70.3, 70.3, 69.6, 68.4, 68.4, 68.1, 68.1, 65.4, 50.9, 50.9, 35.5, 31.5.

Section C. NMR Spectroscopy

a) NMR Spectra in CD_3CN:

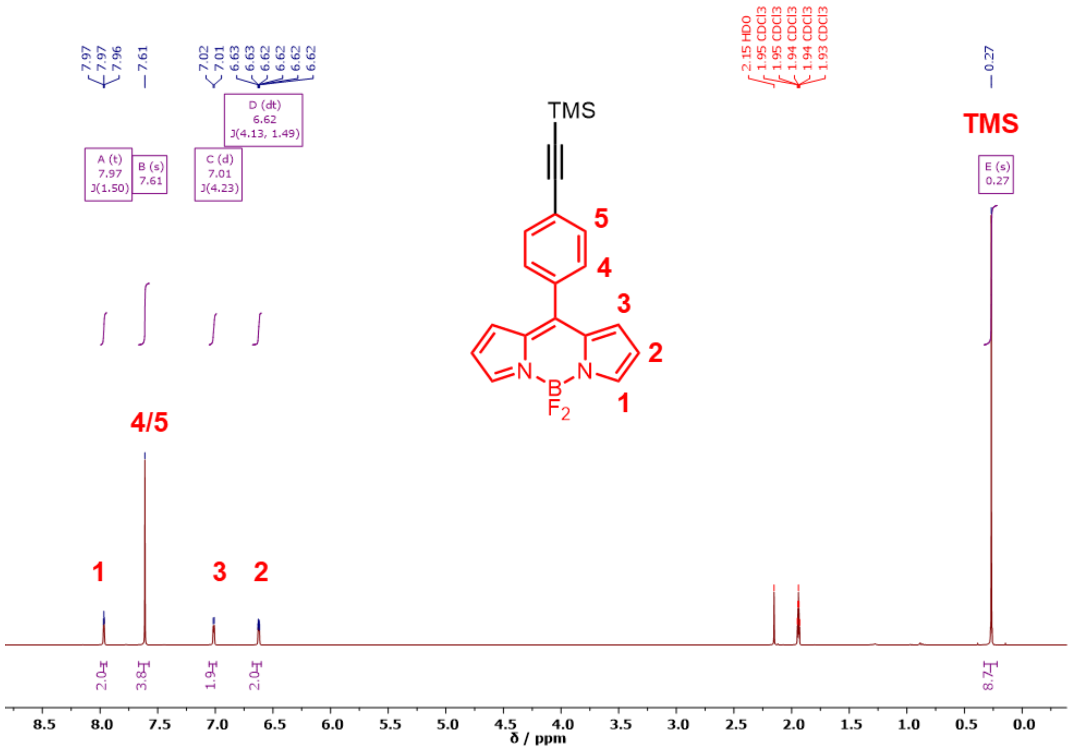

Figure S1. ^{1}H NMR Spectrum (500 MHz, CD_3CN) of **3**.

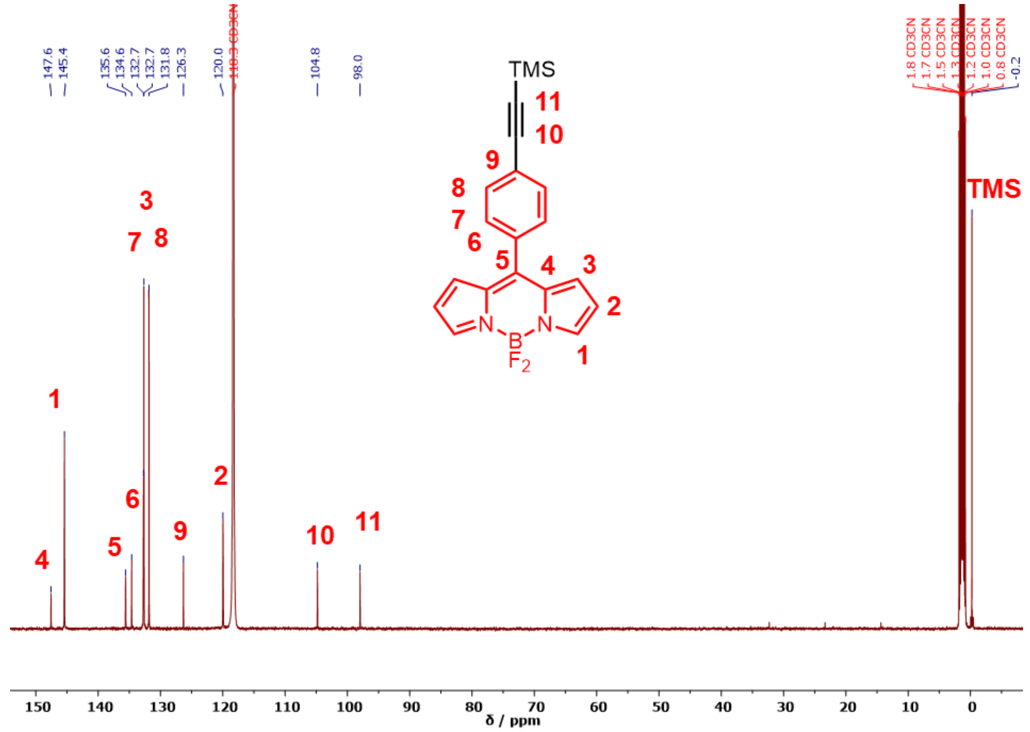

Figure S2. ^{13}C NMR Spectrum (125 MHz, CD_3CN) of **3**.

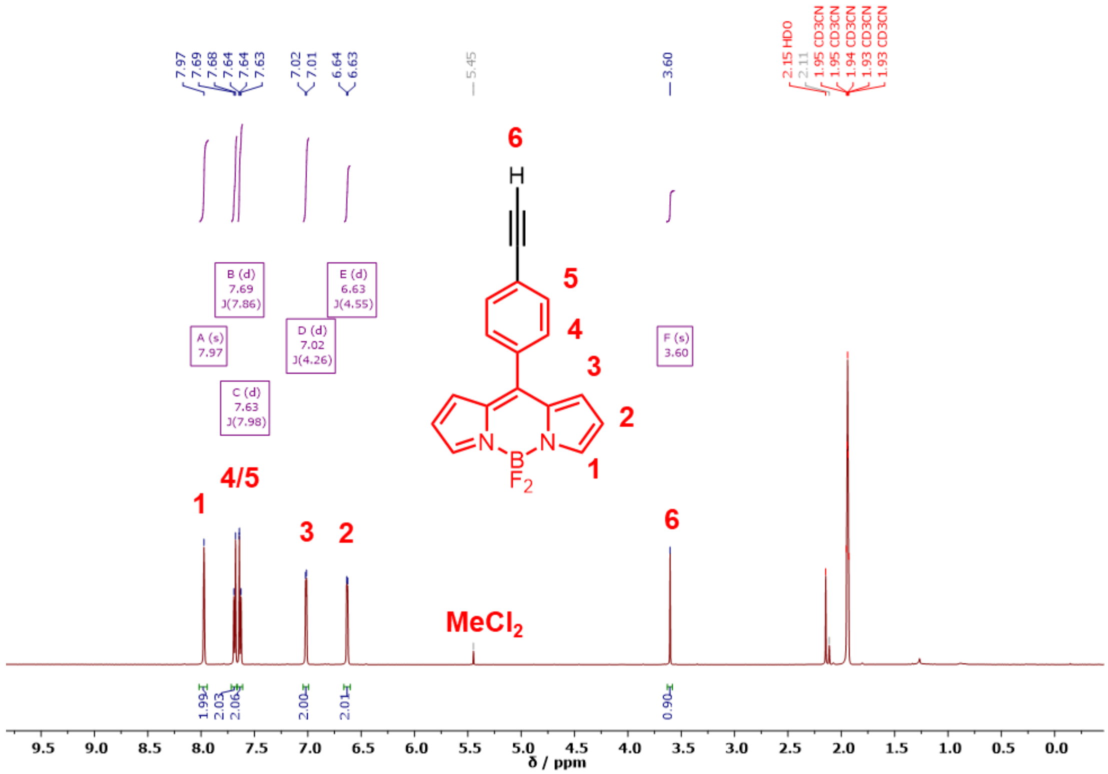

Figure S3. ^{1}H NMR Spectrum (500 MHz, CD_3CN) of **4**.

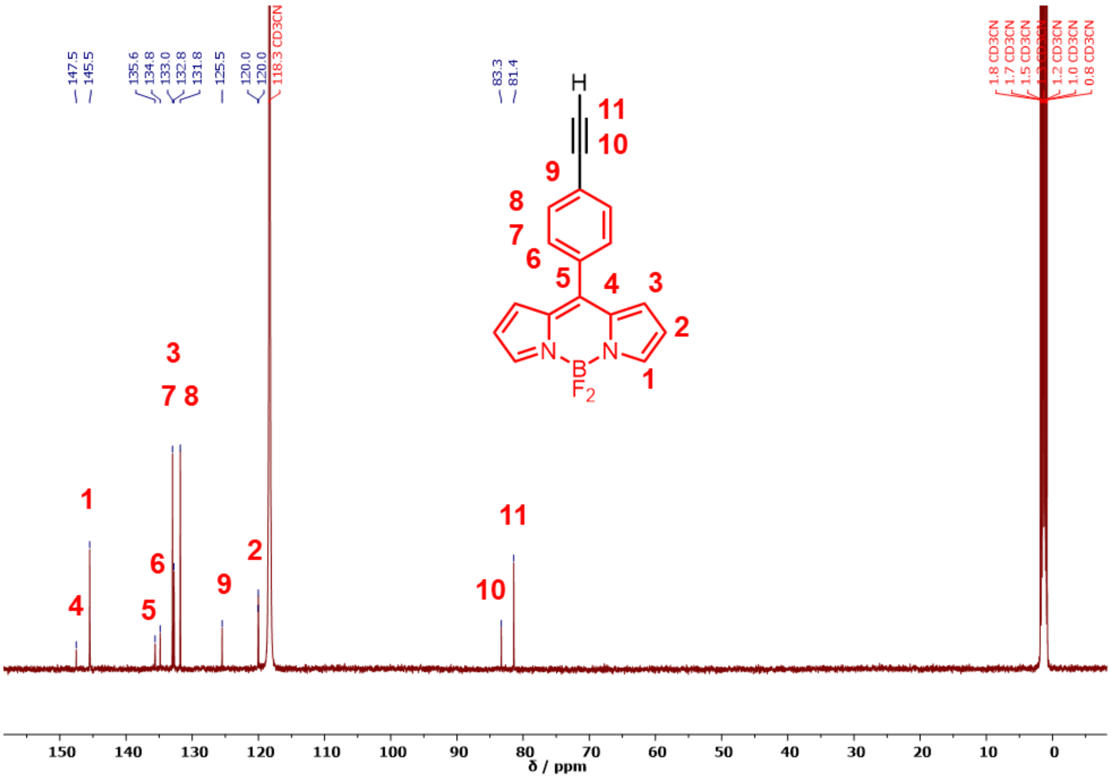

Figure S4. ^{13}C NMR Spectrum (125 MHz, CD_3CN) of **4**.

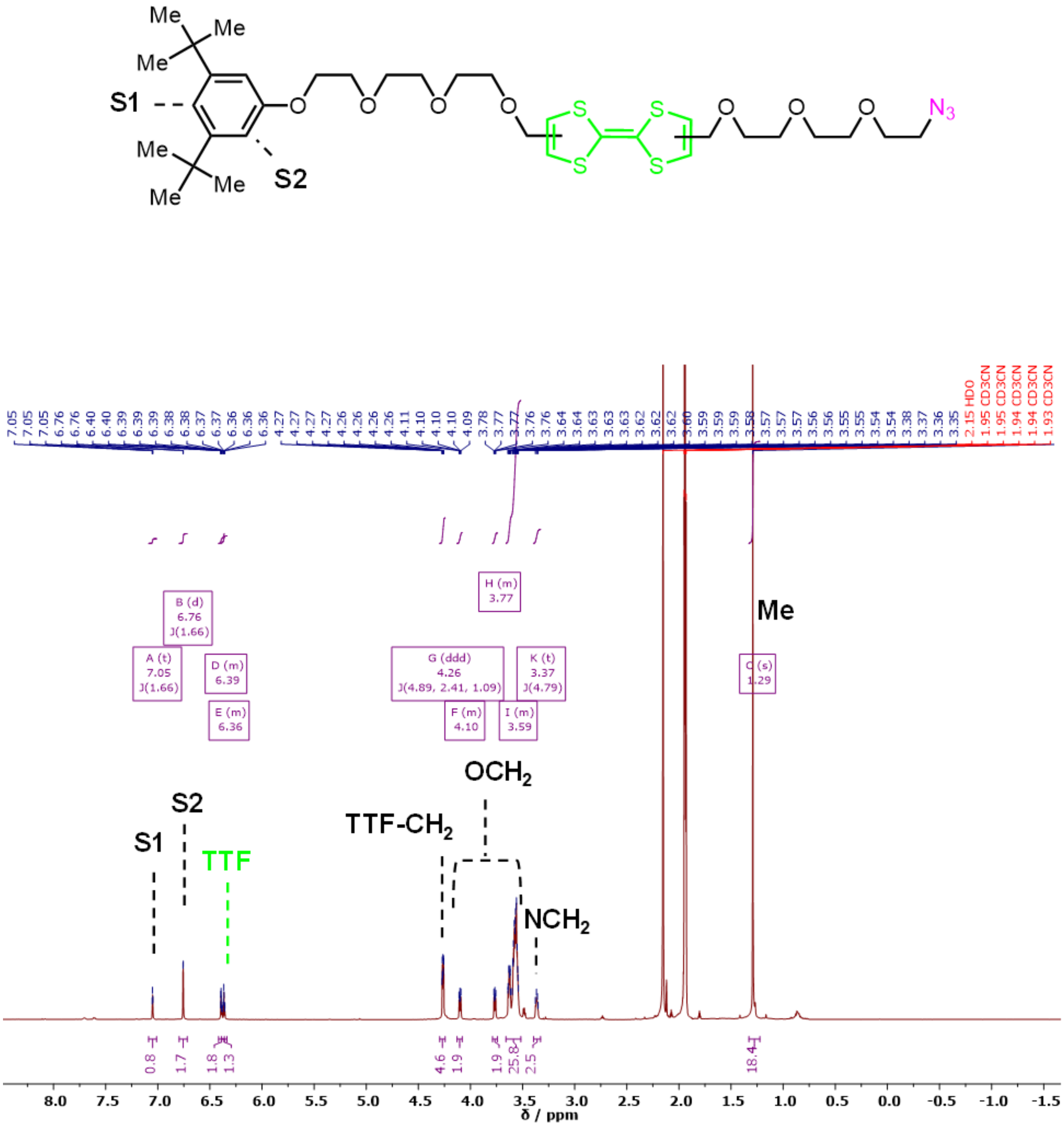

Figure S5. ^{1}H NMR Spectrum (500 MHz, CD_3CN) of **5**.

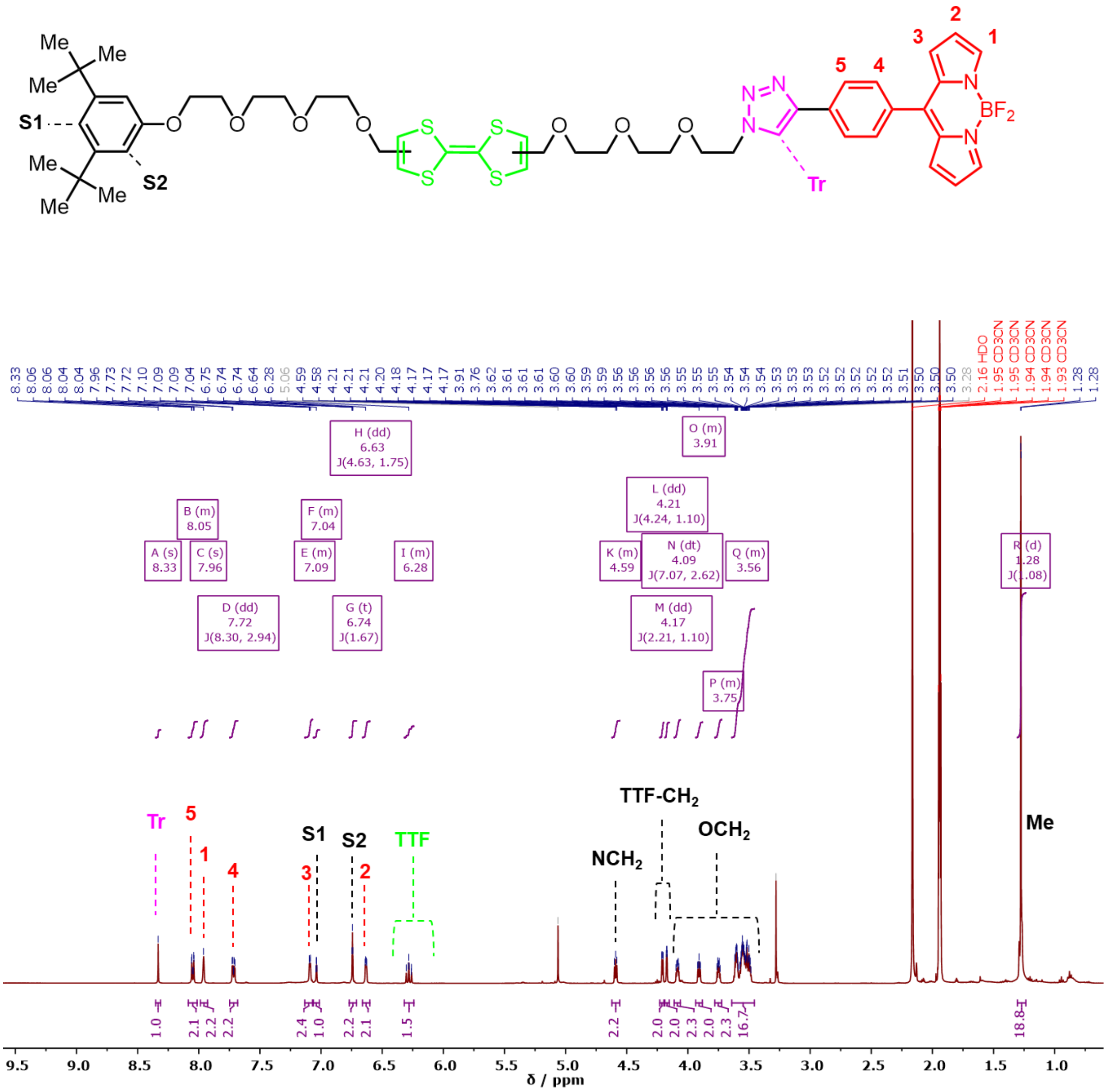

Figure S6. ^{1}H NMR Spectrum (500 MHz, CD_3CN) of **DB**.

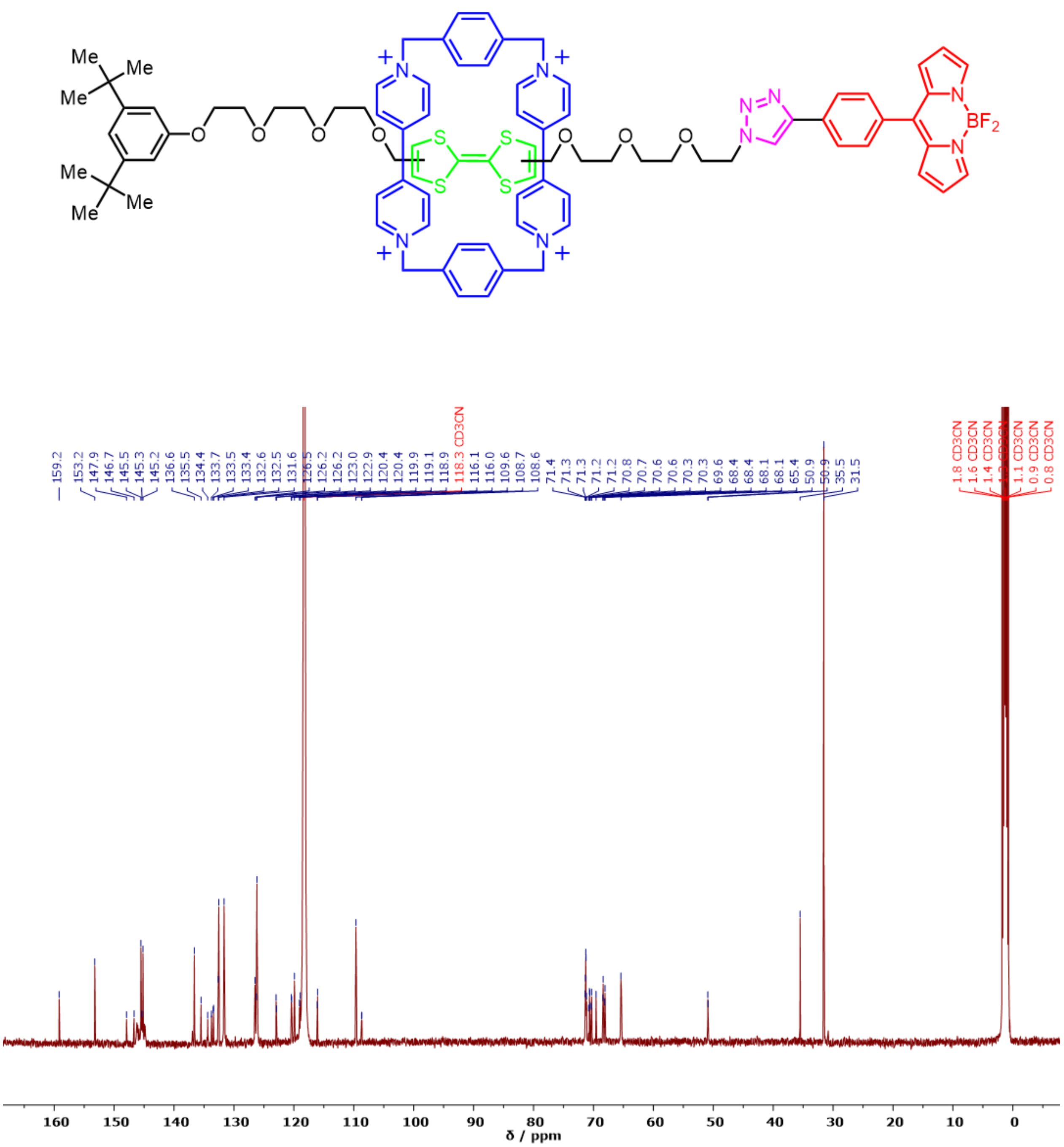

Figure S7. ^{13}C NMR Spectrum (125 MHz, CD_3CN) of **6**•$4PF_6$.

***b)* VT-NMR Spectra in CD3CN:**

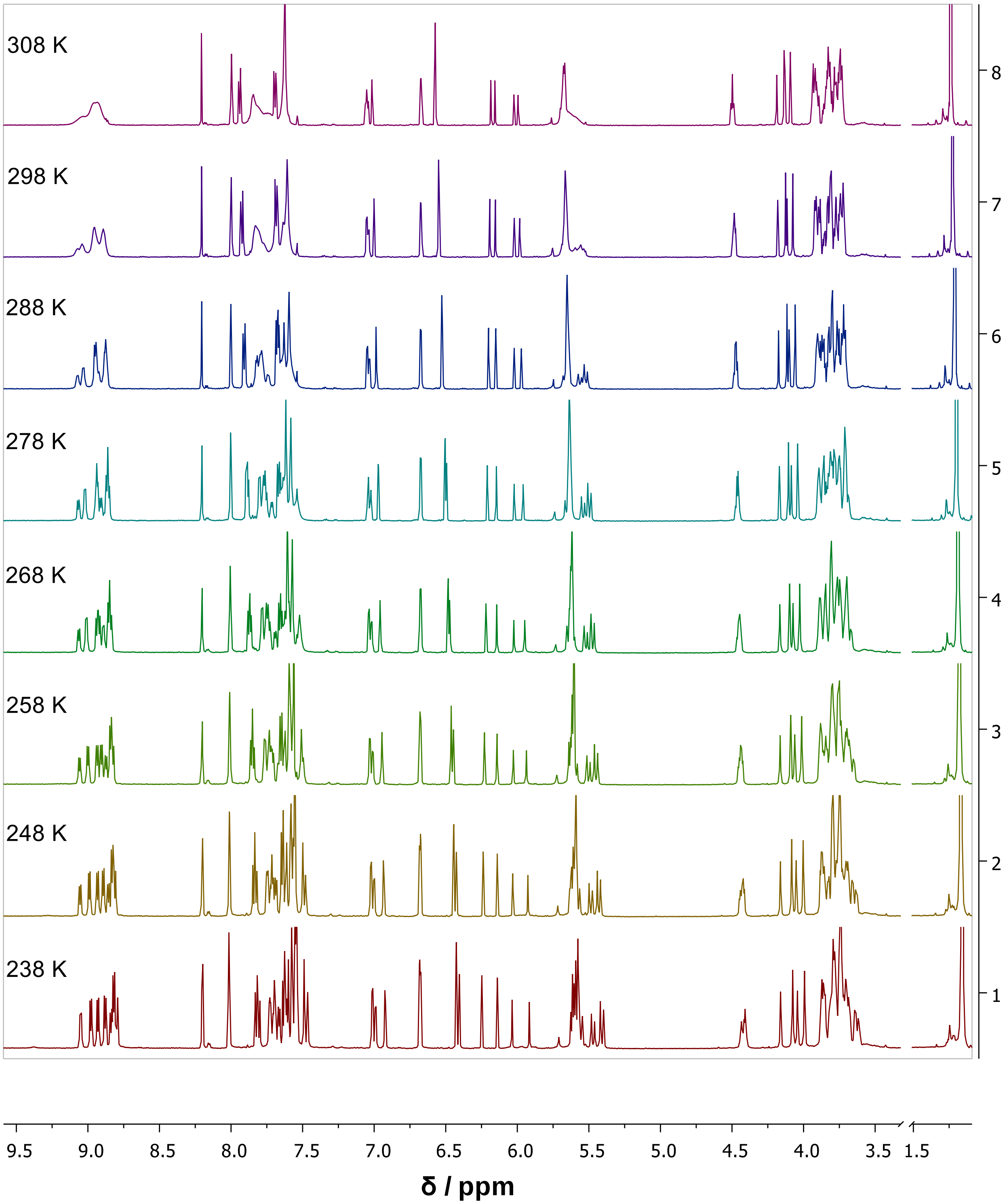

Figure S8. Variable-Temperature ^{1}H NMR Spectrum (500 MHz, CD_3CN) of **6**•4PF_6.

c) **2D-NMR Spectra in CD3CN:**

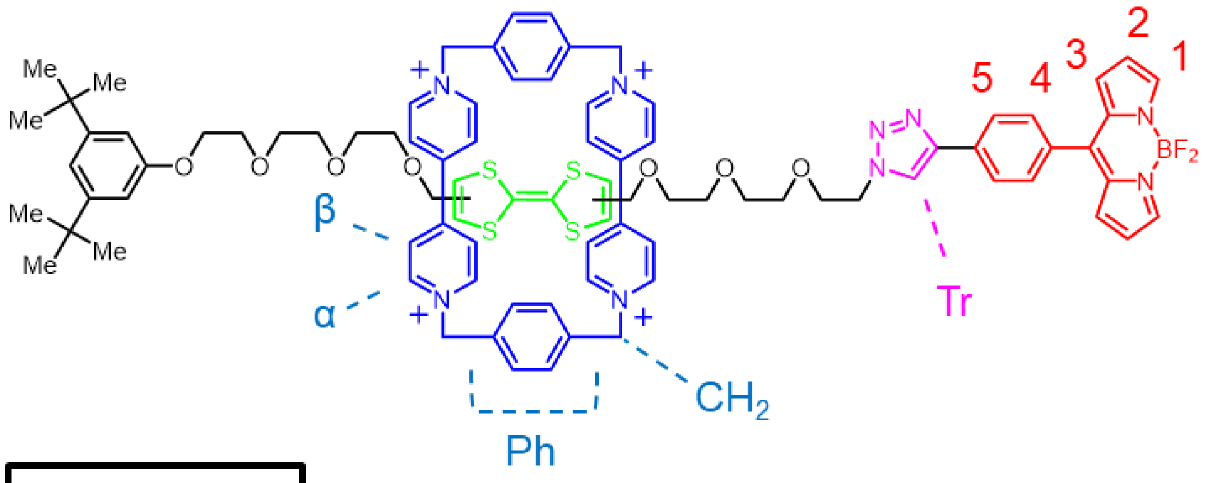

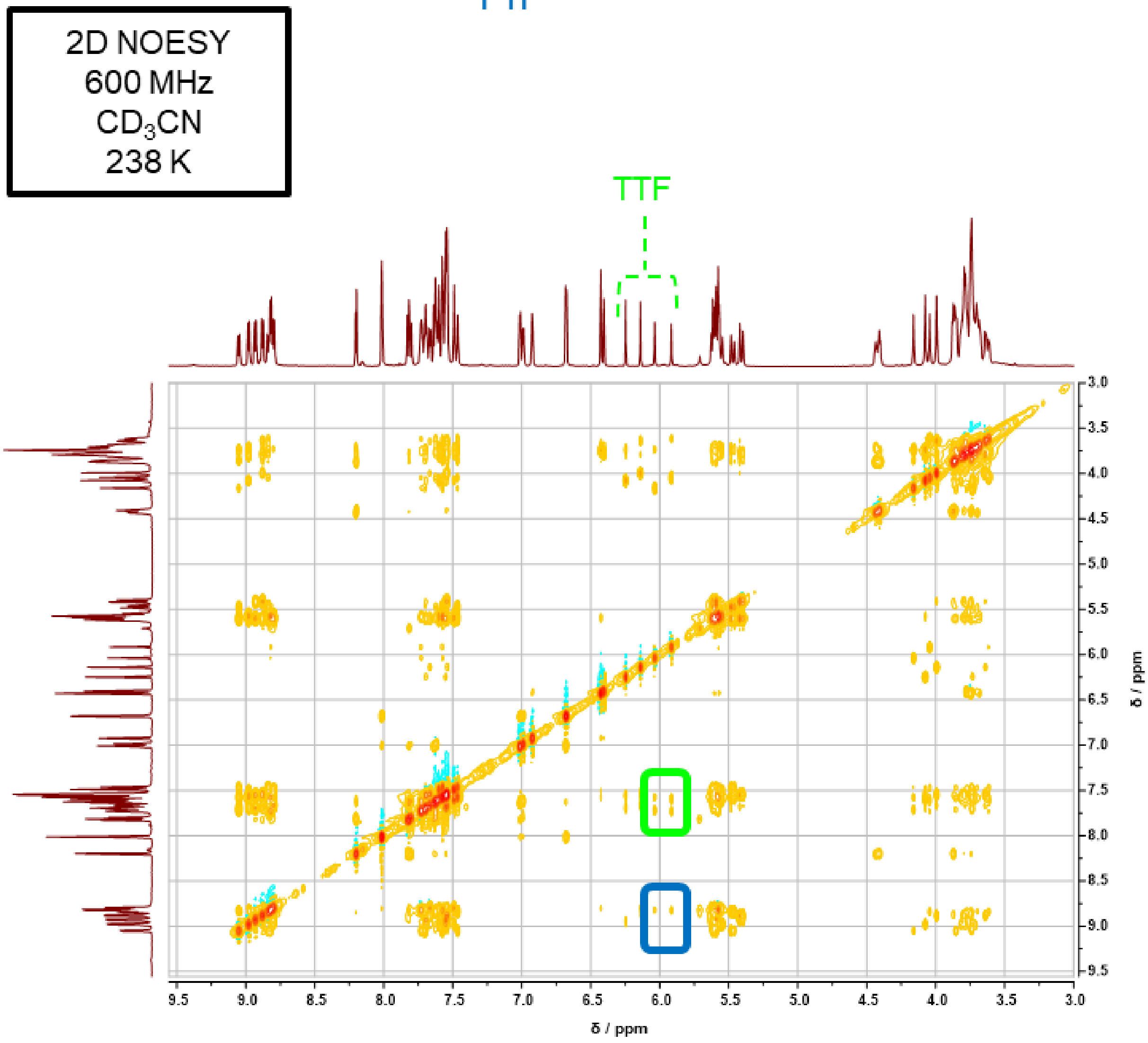

Figure S9. ^{1}H-^{1}H 2D-NOESY Spectrum (500 MHz, CD_3CN, 238 K) of **6**•4PF_6.

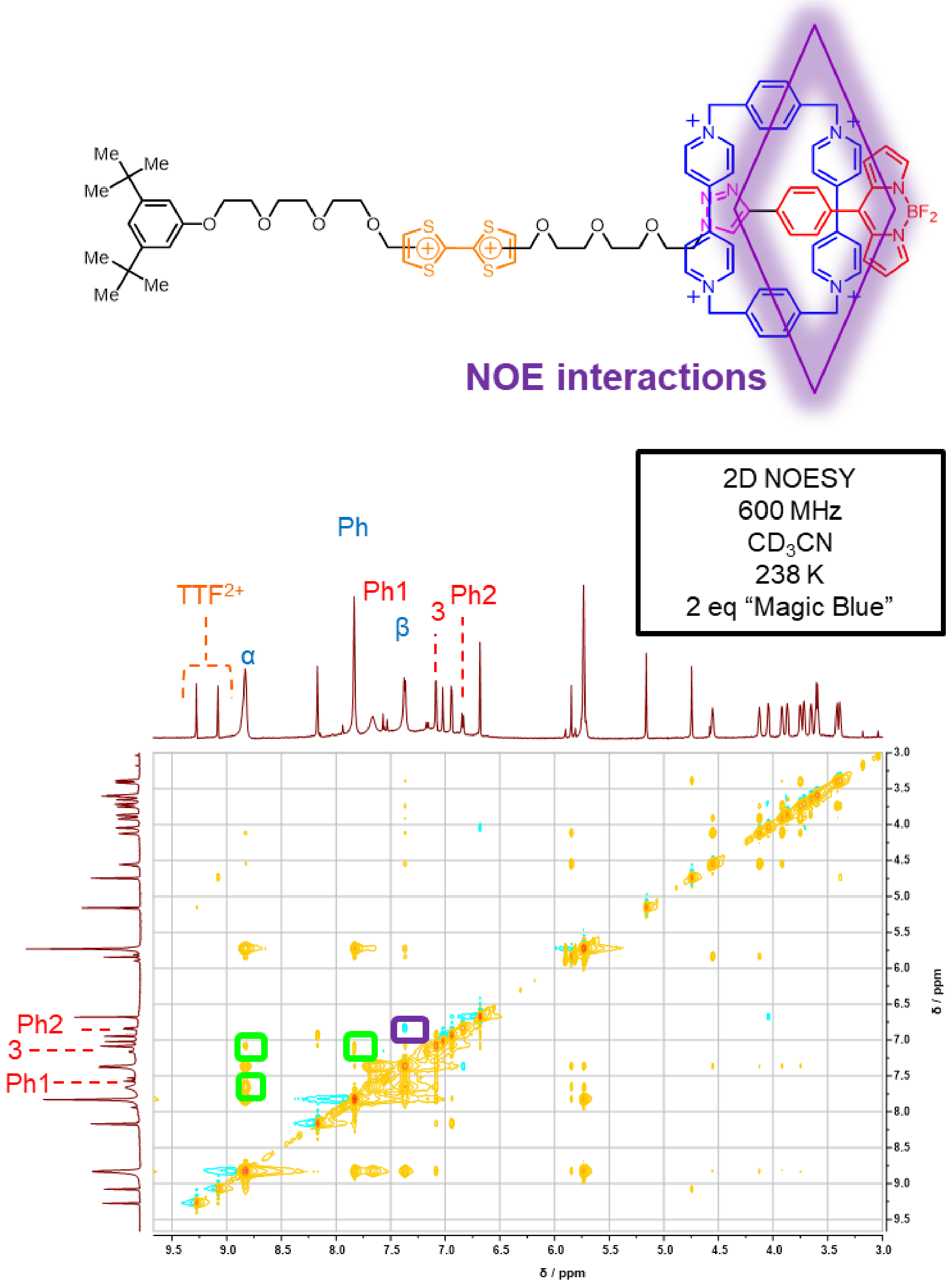

Figure S10. ^{1}H-^{1}H 2D-NOESY Spectrum (500 MHz, CD_3CN, 238 K) of **6**•$4PF_6$ chemical oxidation by addition of "Magic Blue".

Section D. Electrochemistry

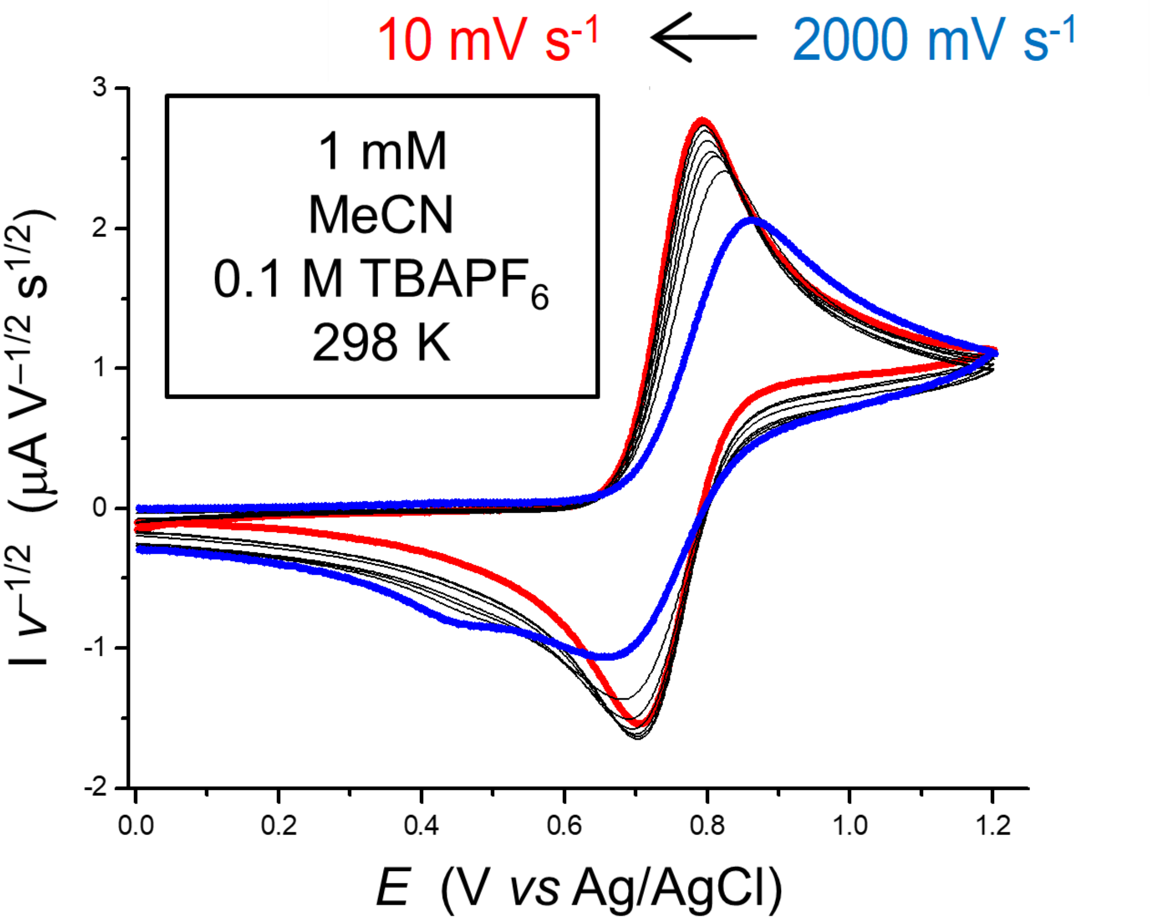

Figure S11. Cyclic voltammograms of [2]rotaxane **6**•4PF_6 at different scan rates.

Section E. Fluorescence Spectroscopy

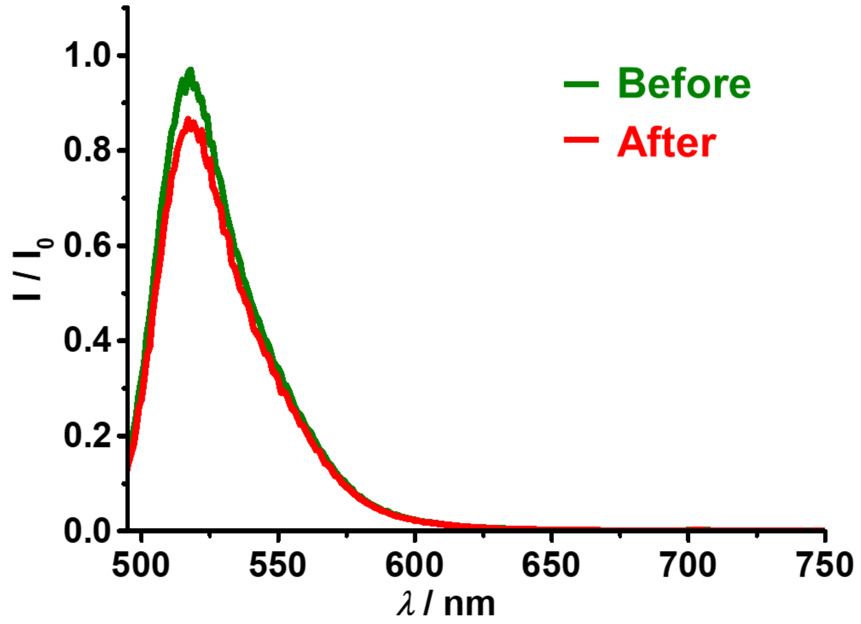

Figure S12. Fluorescence signal of the BODIPY unit in the reference dumbbell DB measured before and after oxidation of the TTF unit showing no appreciable fluorescence changes.

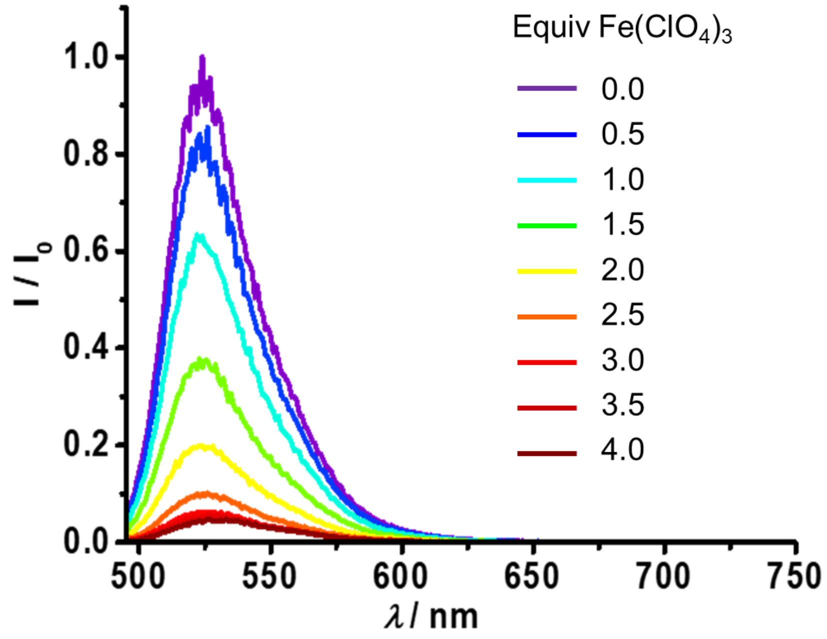

Figure S13. Fluorescence signal changes of the bistable [2]rotaxane **6**•4PF_6 in PhMe:MeCN (90:10 vol/vol) binary mixture following addition of $Fe(ClO_4)_3$ as the chemical oxidant.

Section F. Femtosecond Transient Absorption (fsTA) Spectroscopy

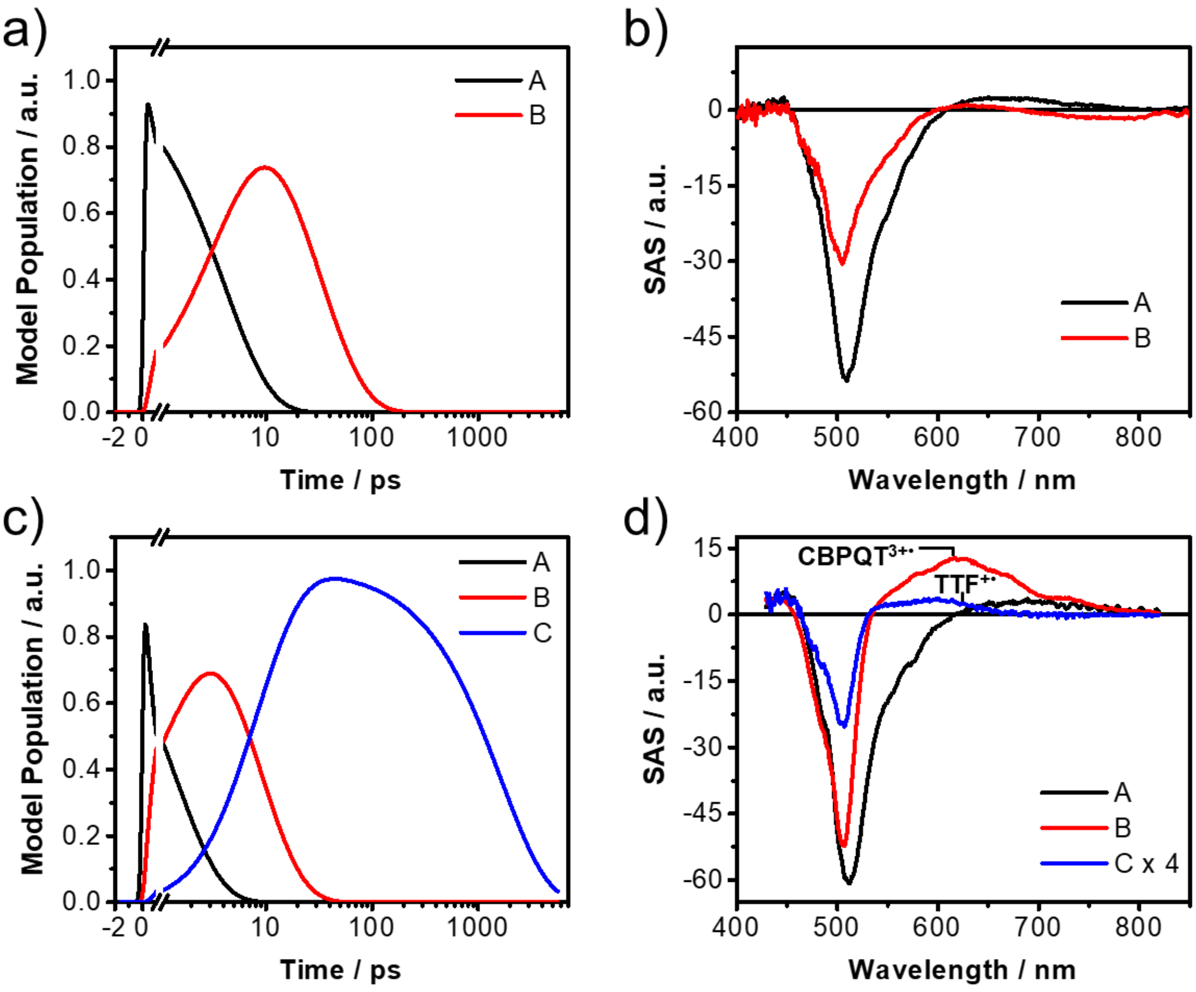

Figure S14. a,c) Kinetic model population distributions and b,d) species-associated spectra of the fsTA spectroscopy of the bistable [2]rotaxane **6**•$4PF_6$ in MeCN a-b) before and c-d) after addition of 2 equiv of $Fe(ClO_4)_3$ as the chemical oxidant.

Section G. Quantum Mechanical Calculations

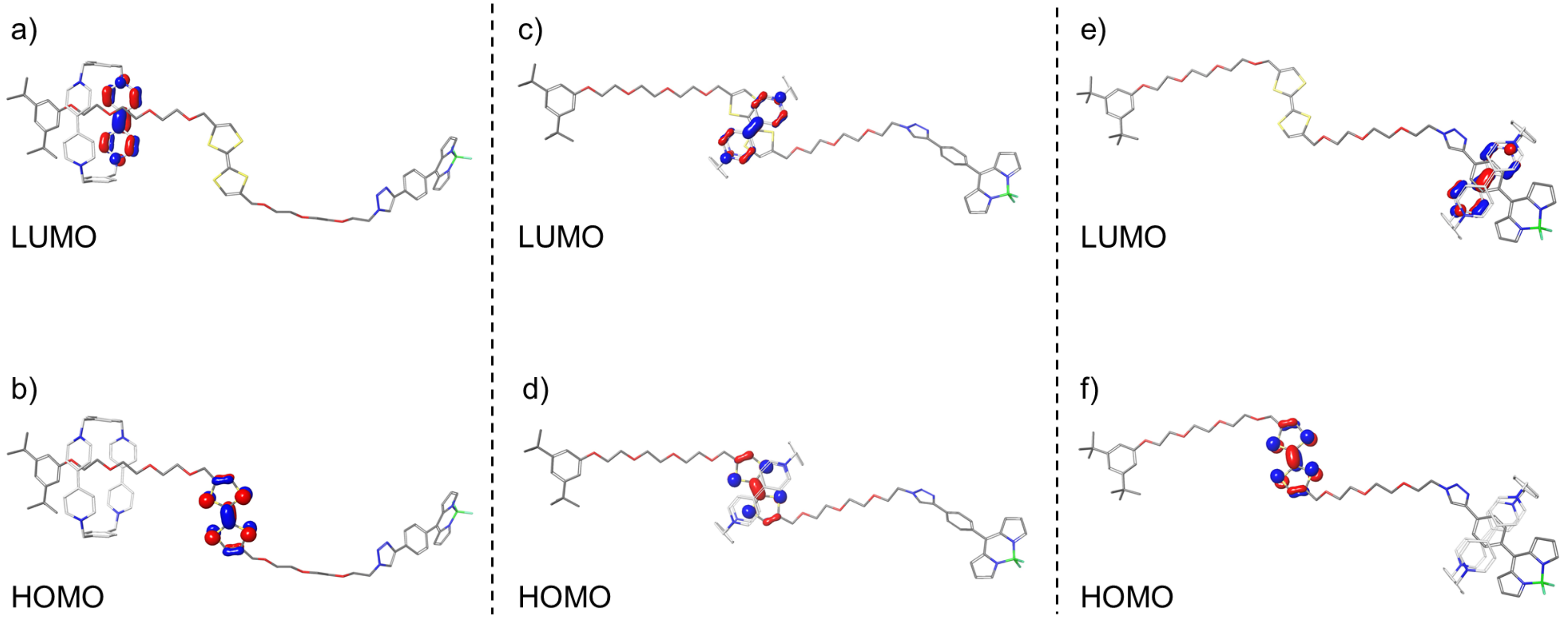

Figure S15. DFT Calculated ground-state structures and relevant frontier orbitals of $\mathbf{6}^{4+}$, where the $CBPQT^{4+}$ ring resides on a-b) di-*t*-butyl benzene side, on c-d) TTF unit, and on e-f) BODIPY rotor.

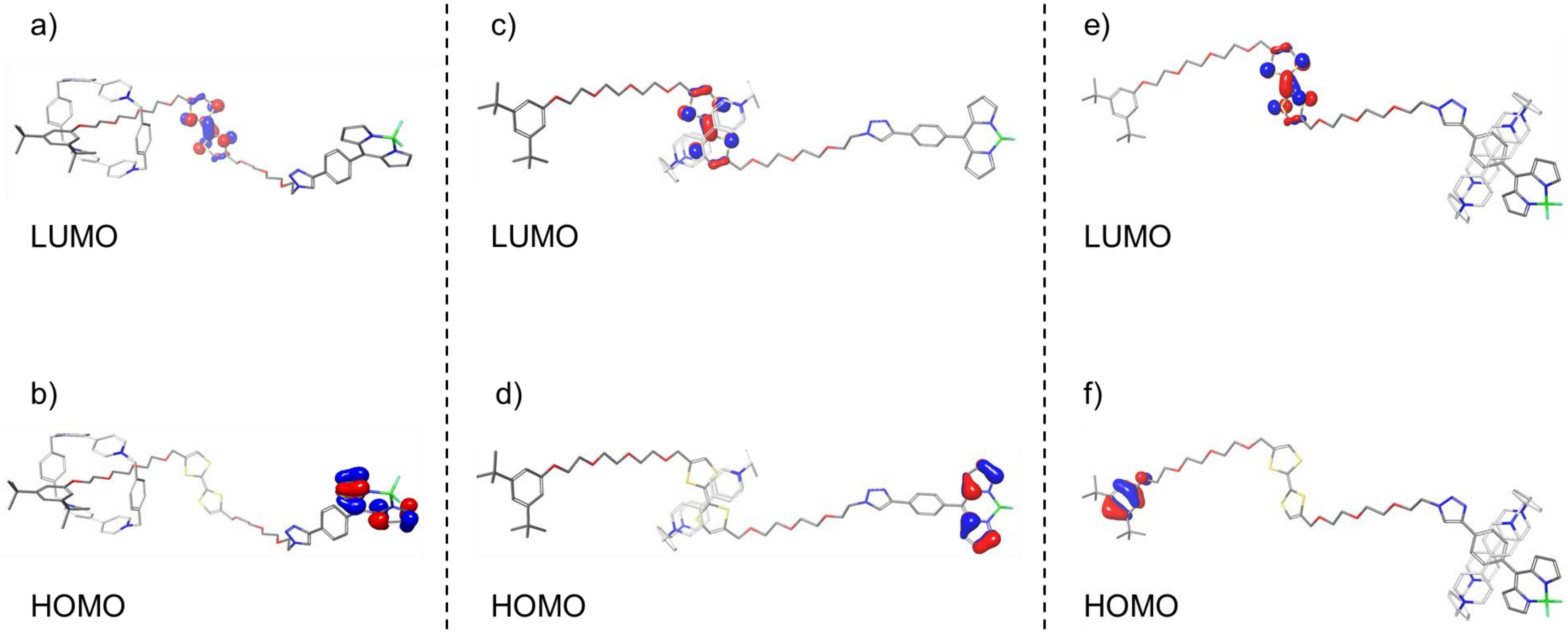

Figure S16. DFT Calculated ground-state structure and relevant frontier orbitals of **6**$^{6+}$, where the CBPQT^{4+} ring resides on a-b) di-*t*-butyl benzene side, on c-d) TTF unit, and on e-f) BODIPY rotor.

Section H. References

S1 Avellini, T.; Li, H.; Coskun, A.; Barin, G.; Trabolsi, A.; Basuray, A. N.; Dey, S. K.; Credi, A.; Silvi, S.; Stoddart, J. F.; Venturi, M. Photoinduced Memory Effect in a Redox Controllable Bistable Mechanical Molecular Switch. *Angew. Chem. Int. Ed.* **2012**, *51*, 1611–1615.
